# Iodine Atoms: A New Molecular Feature for the Design of Potent Transthyretin Fibrillogenesis Inhibitors

**DOI:** 10.1371/journal.pone.0004124

**Published:** 2009-01-06

**Authors:** Teresa Mairal, Joan Nieto, Marta Pinto, Maria Rosário Almeida, Luis Gales, Alfredo Ballesteros, José Barluenga, Juan J. Pérez, Jesús T. Vázquez, Nuria B. Centeno, Maria Joao Saraiva, Ana M. Damas, Antoni Planas, Gemma Arsequell, Gregorio Valencia

**Affiliations:** 1 Unit of Glycoconjugate Chemistry, Institut de Química Avançada de Catalunya, I.Q.A.C.-C.S.I.C., Barcelona, Spain; 2 Laboratory of Biochemistry, Institut Químic de Sarrià, Universitat Ramon Llull, Barcelona, Spain; 3 Departamento de Ingenieria Química, ETSEIB-Universitat Politècnica de Barcelona, Barcelona, Spain; 4 IBMC - Instituto de Biologia Molecular e Celular, Universidade do Porto, and ICBAS - Instituto de Ciências Biomédicas Abel Salazar, Universidade do Porto, Porto, Portugal; 5 Instituto Universitario de Química Organometálica “Enrique Moles”, Unidad Asociada al C.S.I.C. Universidad de Oviedo, Oviedo, Spain; 6 Instituto Universitario de Bio-Orgánica “Antonio González”, Universidad de La Laguna, La Laguna, Tenerife, Spain; 7 Computer-Assisted Drug Design Laboratory, Research Group on Biomedical Informatics (GRIB) IMIM-Universitat Pompeu Fabra, Barcelona, Spain; Swiss Federal Institute of Technology Lausanne, Switzerland

## Abstract

The thyroid hormone and retinol transporter protein known as transthyretin (TTR) is in the origin of one of the 20 or so known amyloid diseases. TTR self assembles as a homotetramer leaving a central hydrophobic channel with two symmetrical binding sites. The aggregation pathway of TTR into amiloid fibrils is not yet well characterized but *in vitro* binding of thyroid hormones and other small organic molecules to TTR binding channel results in tetramer stabilization which prevents amyloid formation in an extent which is proportional to the binding constant. Up to now, TTR aggregation inhibitors have been designed looking at various structural features of this binding channel others than its ability to host iodine atoms. In the present work, greatly improved inhibitors have been designed and tested by taking into account that thyroid hormones are unique in human biochemistry owing to the presence of multiple iodine atoms in their molecules which are probed to interact with specific halogen binding domains sitting at the TTR binding channel. The new TTR fibrillogenesis inhibitors are based on the diflunisal core structure because diflunisal is a registered salicylate drug with NSAID activity now undergoing clinical trials for TTR amyloid diseases. Biochemical and biophysical evidence confirms that iodine atoms can be an important design feature in the search for candidate drugs for TTR related amyloidosis.

## Introduction

Alzheimer's disease is the best example of the 20 or so known amyloid diseases, in which protein or peptidic aggregates are considered to be the direct or indirect origin of the pathological conditions of the disease [1], [2], [3]. A distinctive group of diseases where amyloid deposition does not mainly occur in the central nervous system but rather in several organs in the periphery is associated to the plasma protein transthyretin (TTR). Amyloidosis linked to wild type TTR appears to cause senile systemic amyloidosis (SSA) [4], [5], whereas most of the one hundred TTR mutants, already identified, result in familial amyloidotic polyneuropathy (FAP) [6], [7].

TTR binds and transports 15–20% of serum thyroxine (T_4_) and up to 80% of thyroxine in central nervous system [8]. In addition, TTR is the main carrier of vitamin A by forming a complex with retinol-binding protein (RBP) [9]. To physiologically function, the TTR molecule is self-assembled as a homotetramer, leaving a central hydrophobic channel with two symmetrical binding sites [10], [11].

Recent studies on the aggregation pathway of TTR into amyloid fibrils point to a fibrillogenesis model which involves several steps such as dissociation of the tetramer, changes on monomer conformation, aggregation of conformationally modified monomers into non-fibrillar oligomers that latter form protofibrils and further elongate into mature fibrils [12]–[15]. This mechanism along with the fact that binding of thyroid hormones to TTR results in tetramer stabilization, suggests that inhibition of amyloid fibril formation can be accomplished by small molecule compounds [16]–[26] sharing structural similarities with T_4_. Indeed this hypothesis has been confirmed by the identification of several families of compounds that, by binding to TTR, stabilize the ground state of the protein to an extent which is proportional to the dissociation constants [27]. The most common molecular features on this range of inhibitors [28]–[43] is that they are composed of two aromatic rings bearing halogen substituents in one moiety and hydrophilic functions in the second which give rise to structures as diverse as tetrahydroquinolines, dihydropyridines, benzodiazepines, phenoxazines, stilbenes and benzoxazoles [44], [45].

**Figure 1:**
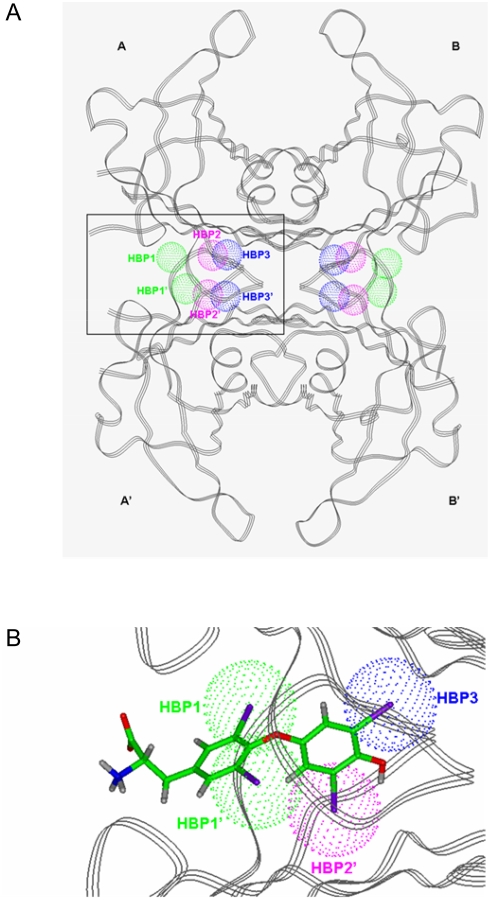
A) Ribbon diagram of the quaternary structure of TTR with a schematic representation of the three-related pairs of pockets capable of accommodate an iodine atom in each binding site located at the interface of monomers A–A′ and B–B′. These pockets are named in the literature HBP1-HBP1′ (green spheres), HBP2-HBP2′ (pink spheres) and HBP3-HBP3′ (blue spheres). B) Detailed view of one of the binding sites for the TTR:T_4_ complex, showing the occupation of four of the six HBPs by the iodine atoms of T_4_ .

Thyroid hormones are the only human biochemicals presenting multiple iodine atoms in their molecules. Blake and co-workers were the first to describe that in each TTR binding site there are six pockets capable of accomodate an iodine atom (Figure 1a). Indeed, when T_4_ binds TTR, four of these six pockets become occupied by the iodine atoms of the hormone molecule resulting in a close steric fit between the ligand and the binding site (Figure 1b). Therefore, iodine atoms are crucial for the binding mode of thyroid hormones to TTR, making an important contribution to the protein-hormone interactions that stabilise the complex [46]. In spite of this evidence, up to our knowledge, none of the potential newly designed TTR amyloid inhibitors have taken advantage of the potential benefits of incorporating iodine atoms to mimick the iodine-assisted binding mode of thyroid hormones. Accordingly, the aim of the present investigation was to provide initial evidences for the hypothesis that iodine atom addition to already known TTR inhibitors could produce more potent TTR fibrillogenesis inhibitors (hereafter referred to as the iodination hypothesis).

Salicylates look particularly interesting as drug candidates due to their long therapeutic tradition and wide clinical applications. Owing that a number of salicylate analogues have also been postulated as good TTR amyloid inhibitors and because the salicylic core is amenable to electrophilic iodination, a salicylate was chosen as a model template to test this hypothesis. Among the many posible analogues a difluorophenyl derivative, namely, diflunisal (2′,4′-difluoro-4-hydroxy-[1,1′-biphenyl]-3-carboxylic acid) was selected since it is an already registered drug [47] having a biphenyl core structure which complies with the two-ring model of TTR inhibitors shows a good TTR amyloid inhibitory profile [48]–[52], and is under clinical trials for TTR-related amyloidosis [53].

## Results and Discussion

### Computational GRID studies of TTR binding site

Naturally occurring TTR is composed of four chemically identical monomers folded in a ß sandwich arquitecture leaving a central channel where two ligand molecules may bind simultaneously (Figure 1). Owing to the two fold crystallographic axis that runs through this channel there are two symmetry related positions for the ligand at both ends of the channel. As already said, three symmetry related pairs of HBPs capable to accomodate iodine atoms is the most prominent structural feature of this channel.

**Figure 2:**
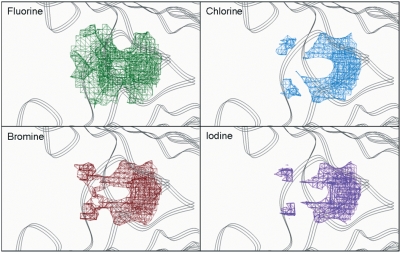
Affinity grids for the different halogens in TTR binding site. Contour maps are drawn at the highest level in which the HBP regions are showed: −0.8 kcal/mol for fluorine (upper left), −2.8 kcal/mol for chlorine (upper right), −3.6 kcal/mol for bromine (bottom left) and −5.2 kcal/mol for iodine (botom right). The contouring of the maps has been done at different levels of energy to emphasize that que energy interaction values increases with the atomic number of the halogen.

To computationally analyze these HBPs we have performed calculations for imaging the grids of affinity between different halogen atom probes and the surfaces of the binding channel. The contour maps of Figure 2 show specific regions with high affinity for all the halogen atoms. The situation of these areas perfectly agrees with the initial geometrical description of HBPs. Their extension is almost identical for every halogen although the close proximity of HBP2 and HBP3 results in a continuous zone with two optimal affinity points matching HBP2 and HBP3. In spite of sharing the same regions, the energy of interaction for every halogen atom is different and its magnitude increases with the atomic number up to a maximum value for iodine (Table 1).

**Table 1:** Energy values (in kcal/mol) of the minima points of affinity grids regions corresponding to HBPs in the A–A′ binding site of TTR:T_4_ complex after removing T_4_ and water molecules (see materials and methods section for details).

	HBP1-HBP1′	HBP2-HBP2′	HBP3-HBP3′
F	−1.3	−3.2	−5.6
Cl	−3.1	−6.7	−7.1
Br	−5.0	−8.5	−9.6
I	−6.2	−9.4	−11.4

### Synthesis of diflunisal analogues

**Figure 3:**
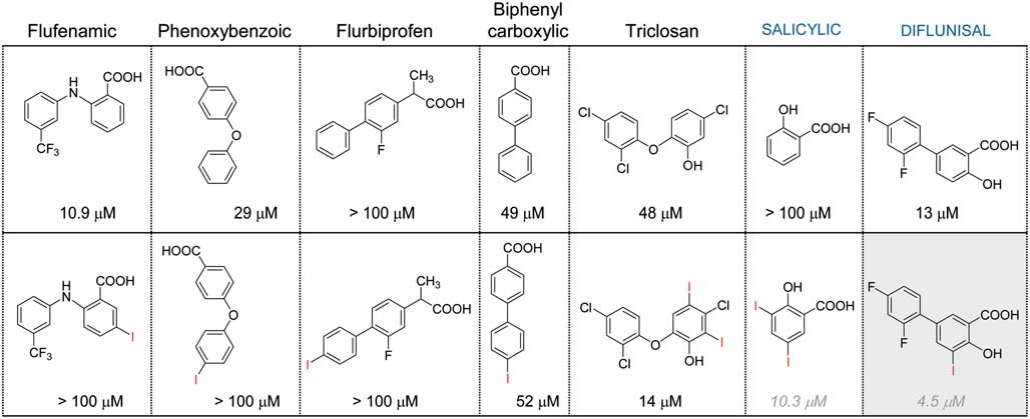
Effect of iodination: proof of concept. Selected families in which iodination enhances inhibitory efficiency.

According to GRID studies, iodine atoms placed at strategic positions of the structure of TTR ligand may maximize their potency by stablishing positive energetic interactions with these high affinity halogen binding regions on the TTR binding channel. To test the iodination hypothesis here proposed, a number of iodinated analogues of already known inhibitors such as, *i.e.*, flufenamic, 4-phenyl and 4-phenoxy benzoic acids have been prepared and tested in our fibrillogenesis inhibition assay. Results from this rough screening (Figure 3) have shown that most striking positive effects on inhibitory potency were found for diflunisal, a FDA-approved cyclooxygenase inhibitor with well documented clinical records as NSAID.

**Figure 4:**
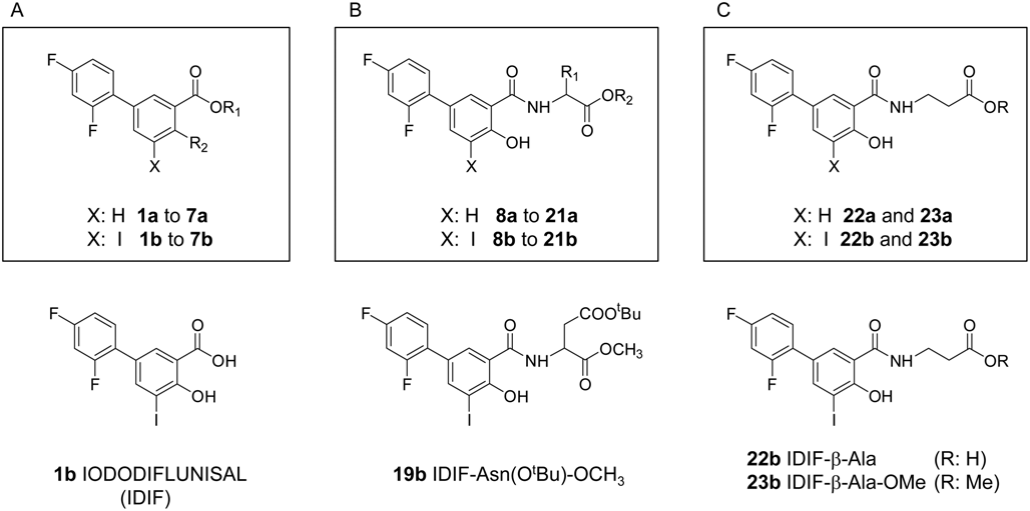
Synthesis of diflunisal and iododiflunisal analogs. A) Modifying functional groups of diflunisal; B) Conjugation to α-amino acids; and C) Conjugation to β-alanine derivatives.

**Figure 5:**
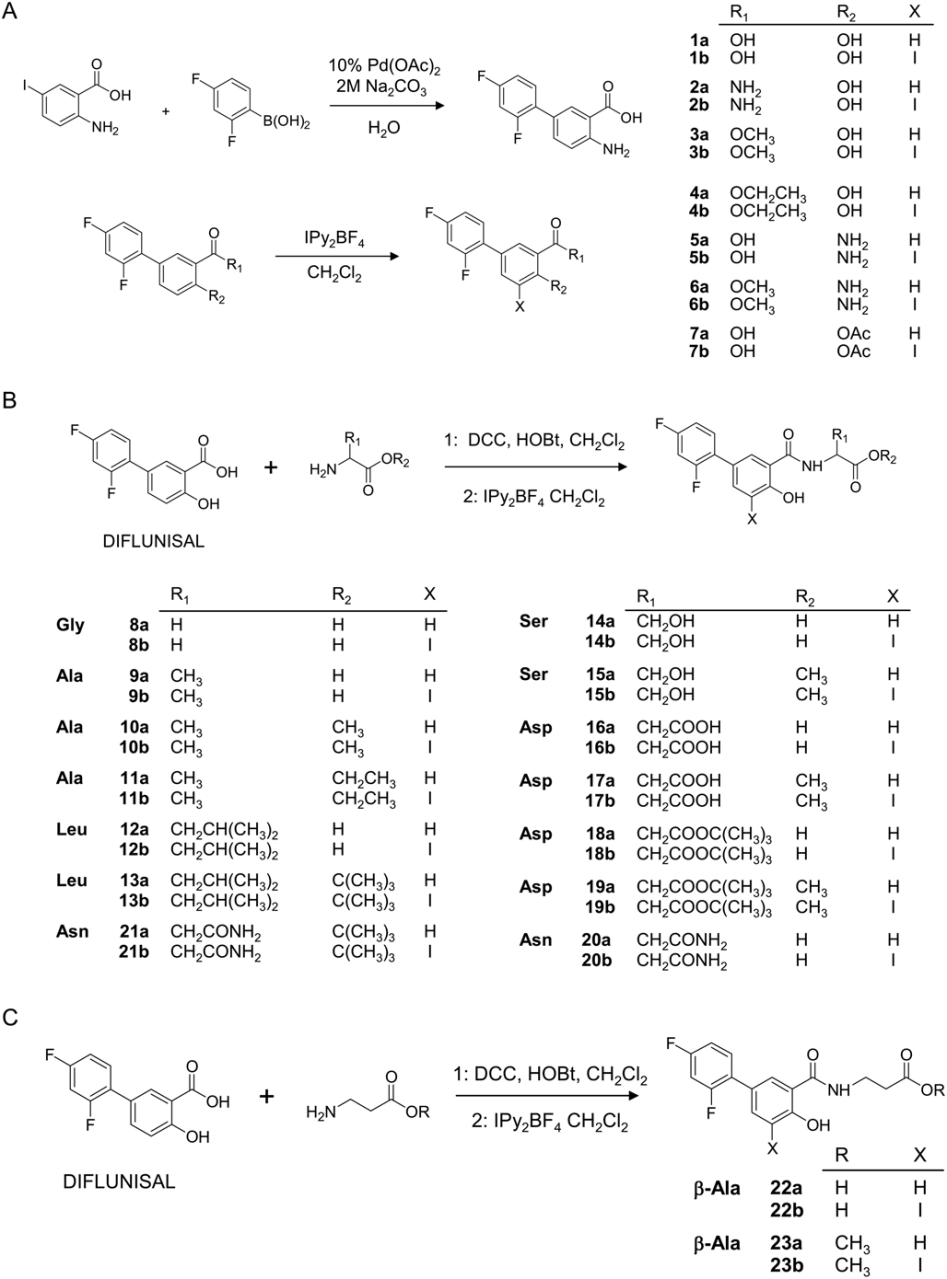
A) Diflunisal analogs: modifications in the salicylic ring; B) Diflunisal conjugates to α-amino acids; C) Diflunisal conjugated to β-alanine.

To further examine how general was this effect on diflunisal analogues, a library of 40 derivatives has been designed and prepared. Two subsets of twin compounds have been synthesized (Figure 4). The products labeled as “**a**” lack iodine atoms while the ones termed as “**b**” show one iodine atom at C-5 position. In turn, both series include two groups of compounds, one prepared by modifiying the functional groups of diflunisal by common organic chemistry reactions (compounds **1a** to **7b**) and a second group originated by conjugation of diflunisal to a series of amino acids by standard peptide synthesis protocols (compounds **8a** to **23b**) (Figure 4 and Figure 5).

### In vitro testing for fibrillogenesis inhibition in a turbidimetric test

**Figure 6:**
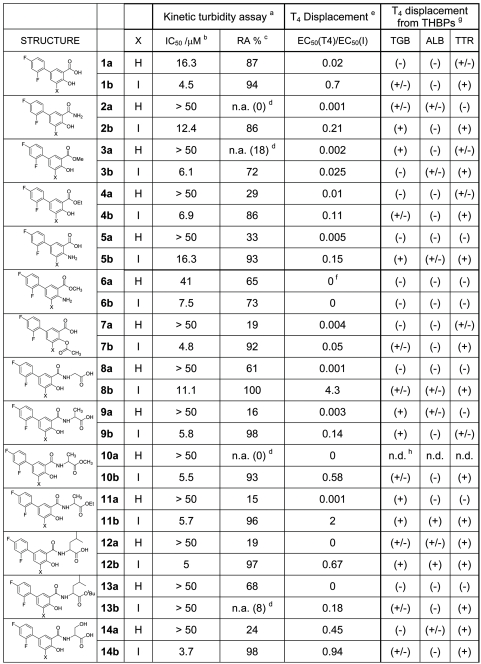
Data from kinetic turbidity assay, T_4_ competition assays and binding selectivity for plasma proteins for compounds 1 to 14 (series a and b).

**Figure 7:**
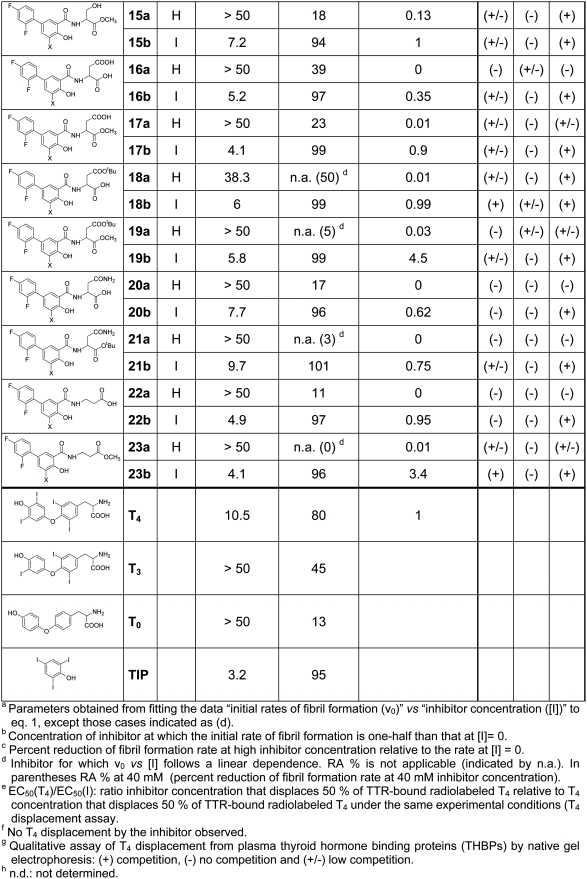
Part II: Data from kinetic turbidity assay, T_4_ competition assays and binding selectivity for plasma proteins for compounds 15 to 23 (series a and b) and reference compounds T_4_, T_3_, T_0_, and TIP.

A high throughput screening assay which measures the ability of individual compounds to inhibit the acid induced fibril formation related turbidity originated by the highly amyloidogenic TTR variant Y78F has been used to assess the *in vitro* amyloid inhibitory properties of these analogues [54]. Two different parameters can be derived from this kinetic test. The IC_50_ value is the inhibitor concentration at which the initial rate of fibril formation is half than that in the absence of inhibitor. In turn, RA (%) can be defined as the percent reduction of fibril formation rate at high inhibitor concentration relative to the rate at zero concentration of tested compound. Values of RA (%) of 100% indicate that the inhibitor is able to fully prevent fibril formation. These inhibition values for the new compounds are given along with the values for the reference compounds, thyroid hormones T_4_ and T_3_, thyronine (T_0_) and triiodophenol (TIP) (Figure 6 and Figure 7).

All the modifications attempted on diflunisal are clearly detrimental and spoil its inhibitory potency (compounds of “**a**” series). However, in most of the cases, the introduction of a iodine atom at C-5 position of the molecule (compounds of “**b**” series) not only recovers the lost potency but many iodinated analogues show good inhibition values approaching the highest record shown by triodophenol (TIP). The same effect is seen with reference compounds: T_0_ (no iodine), T_3_ (3 iodine atoms) and T_4_ (4 iodine atoms), where the presence and load of iodine atoms correlate with potency.

Much of the insoluble material produced in the course of these kinetic tests of aggregation using TTRY78F which are carried out at pH 4.1, is of amyloid nature as checked by optical microscopy after congo red staining. Almost amorphous (non-stainable by congo red) aggregates are obtained when lower pH values (*i.e*., pH = 2.0) are applied to trigger fibrillogenesis. An intermediate situation is seen at higher pH values such as 3.6.

In addition, as transmission electron microscopy (TEM) observations confirm [55], the aggregates thus formed are completely prevented when the turbidimetry assays are performed in the presence of a 1:2 proportion of TTRY78F/iododiflunisal (**1b**). However, similar proportions of diflunisal (**1a**) still allow amyloid precipitates to occur.

### Binding competition between T_4_ and diflunisal analogues

The compounds of the library were further evaluated for their capability to compete with T_4_ for binding to wild type TTR by a gel filtration procedure [55]–[57]. Competition binding experiments using recombinant wild type TTR rendered sigmoidal plots from which EC_50_ values and their corresponding relative ratios (EC_50_ T_4_/EC_50_ Inhibitor) have been derived and listed (Figure 6 and Figure 7).

**Figure 8:**
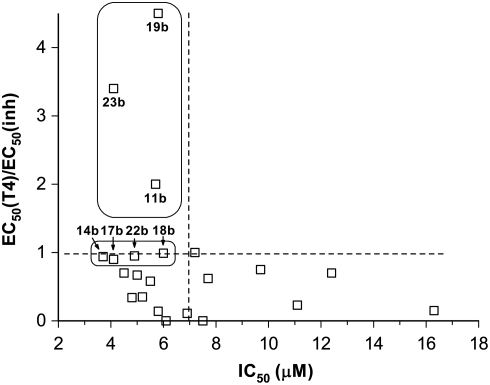
TTR binding *vs.* aggregation inhibition. TTR binding (from T_4_ displacement) *vs.* fibrillogenesis inhibition (from turbidimetric assay) plot for the series of iododiflunisal derivatives 1b–23b. Group 1 and group 2 inhibitors are marked (see text).

Iodinated products are always more potent at displacing T_4_ than their non iodinated counterparts. Striking affinity differences that go up to two and three orders of magnitude can be observed between pairs of compounds such as **1a–b, 8a–b**, **10a–b** and **23 a–b**. In spite of these differences, some relationships can be observed in correlating fibrillogenesis inhibition and relative binding affinity. Thus, by plotting the limits for a reasonably good inhibitor, this is: IC_50_≤7 µM and EC_50_(T_4_)/EC_50_(inh)≥1, in a relative binding affinity *versus* inhibition potency graph, two groups of good inhibitors can be defined (Figure 8). In a first group, the compounds (**11b**, **19b** and **23b)** show higher affinity values for TTR than T_4_ and a common structural feature such as the presence of a carboxylic ester group at 3 to 4 carbon atoms distance from the salicylic ring. In a second group, the binding affinity of the compounds (**14b**, **17b**, **18b** and **22b**) is similar to **T_4_** and a free carboxylic acid function is placed at same distance from the salicylic ring. In addition, the absence of compounds showing high affinity for TTR but low inhibition potency indicate that the fibrillogenesis inhibition properties of these diflunisal analogues arise because of selective binding to the native state of the protein and subsequent kinetic stabilization of its tetrameric form.

### Inhibition rate of TTR tetramer dissociation in 6M urea by diflunisal and iododiflunisal

The stabilizing effect of iodine atoms on the tetrameric form of both TTRwt and TTRY78F has been further evaluted on urea induced dissociation experiments by comparing the performance of iododiflunisal versus diflunisal. Thus, after allowing maximum stabilization of the tetrameric forms of both TTR wild type and Y78F mutant proteins by formation of TTR/inhibitor complexes of (1:2) stoichiometry, measures of circular dichroism in the far-UV of changes in the secondary structure of TTR were recorded as a function of time in 6M urea solutions. It has been assumed that the rate of tetramer dissociation is irreversibly associated to fast monomer unfolding when employing urea concentrations exceeding those that may enable monomer refolding [58].

**Figure 9:**
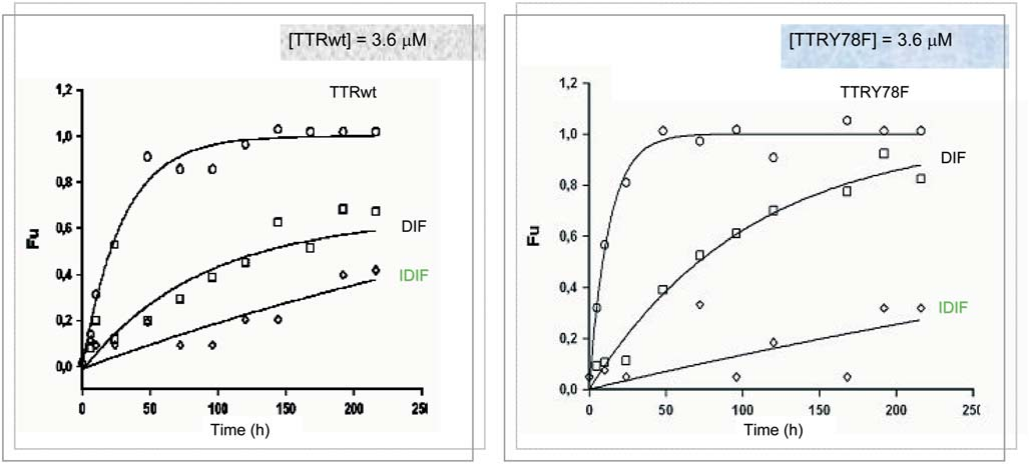
TTR tetramer stabilization by inhibitors monitored by circular dichroism in 6M urea. Influence of small molecule inhibitors diflunisal (1a, 7,2 µM DIF) and iododiflunisal (1b, 7,2 µM IDIF) on the rate of wtTTR (3,6 µM) (left) and mutant Y78FTTR (3,6 µM) (right) dissociation in 6M urea measured by Far-UV CD circular dichroism as a function of time.

Figure 9 shows that the rates of TTRwt and TTRY78F tetramer dissociation in 6M urea proceed at different rates. Such rates are reduced in the presence of diflunisal (**1a**) but more strikingly by iododiflunisal (**1b**). This differential tetramer stabilization that can be attributed to the presence of the iodine atom is in good agreement with similar differences observed from other *in vitro* tests above reported. These results are also consistent with the more potent stabilizing effect seen for iododiflunisal on natural TTR tetramers occurring in blood plasma of control and FAP patients by isoelectric focusing techniques under semi denaturing conditions [55].

### Binding selectivity of compounds for plasma proteins

Binding to TTR is not the exclusive factor in determining the therapeutic potential of new fribrillogenesis inhibitory compounds. Binding specificity to plasma proteins can be a limiting factor for biodistribution, metabolism, activity and toxicity profiles of any potential drug [59]. This is specially crucial in this case because very strong plasma protein competitors of TTR include thyroid binding globulin (TBG), which has an order of magnitude high affinity for thyroxine [60], and albumin (ALB) which is at concentrations of two orders of magnitude higher than TTR in plasma. From our already reported results [55] of binding competition experiments with plasma of transgenic mice and FAP patients, iododiflunisal but not diflunisal has been shown to be a very selective ligand for TTR which is another indication of the important role of the iodine atom. This has been further confirmed by qualitatively assessing the ability of the new compounds to bind to the three T_4_ binding plasma proteins (THBPs), thyroid binding globulin (TBG), albumin (ALB) and TTR. The procedure has been essentially a T_4_ binding displacement experiment but performed using whole human plasma followed by a separation step of the serum proteins by polyacrylamide gel electrophoresis (PAGE).

The two most selective compounds which exclusively bind to TTR (**20b** and **22b**) are iodinated (Figure 6 and Figure 7). One of them (**22b**) is also a very potent inhibitor with equal affinity for TTR than T_4_. A total of 17 less selective compounds show good affinity for TTR (+) and some binding (+/−) for only one of the two proteins TBG or ALB, among them, 13 compounds are iodinated.

### Crystal structures of TTR of two new complexes of TTR with diflunisal analogs (23b and 22b)

Additional evidence for the iodination hypothesis has been sought by elucidating at the atomic level how structural features such as iodination and presence of a carboxyl group enhance the binding affinity of these analogues. Two compounds: **23b** and **22b** were selected for structural studies (Figure 6, Figure 7, and Text S1). One of them, **23b** has been chosen because is one of the most effective T_4_ displacing compounds with a high fibrillogenesis inhibitory potency. The second compound, **22b** is a very closely related analogue lacking a methyl ester group which is also a very potent fibril inhibitor which retains the same binding affinity as T_4_ [EC_50_ (**T_4_**)/EC_50_ (**22b**) = 0.95]. In addition **22b** is one of the two most selective binders of this series of inhibitors when tested in human plasma. Both show very close molecular structures and each one is representative of one of the two differential binding affinity groups discussed above.

The crystal structures of their complexes with TTR could be determined and refined to 1.85 and 1.80 Å and are here compared to the ones of their parent compounds diflunisal and iododiflunisal. While the iododiflunisal-TTR complex has been elucidated by us [61], the diflunisal parameters, here discussed, are taken from published descriptions [52].

**Figure 10:**
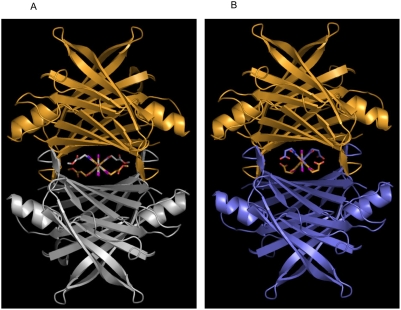
Ribbon diagram of the quaternary structure of TTR in complex with iododiflunisal-betaAlaOMe (23b) (A) and betaAlaOH (22b) (B) conjugates. Because of the two-fold symmetry axis running along the hormone-binding channel there are two-symmetry related binding positions of the two compounds in each binding site.

**Figure 11:**
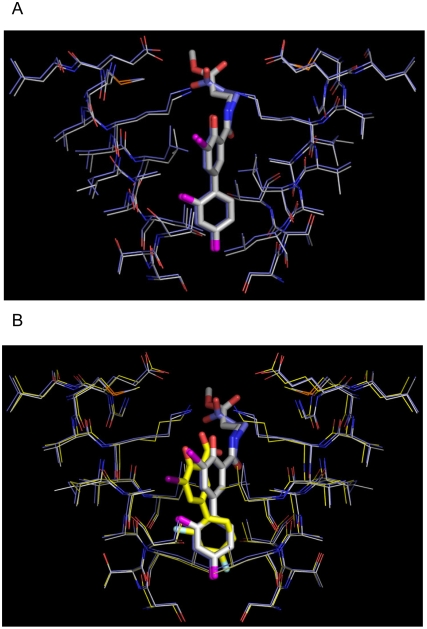
Crystal structures of the TTR-22b and TTR-23b complexes. A) Close-view of the TTR hormone binding sites AA′ of the TTR:iododiflunisal-betaAlaOMe (23b) (in grey) and TTR:iododiflunisal-betaAlaOH (22b) (in blue) superposed. Only one of the two-symmetry related binding positions of these two iododiflunisal conjugates is shown. B) Superposition of the crystal structures of the TTR:iododiflunisal-betaAlaOMe (23b) (in grey), TTR:iododiflunisal-betaAlaOH (22b) (in blue), and TTR:iododiflunisal (1b) complex (in yellow).

The positioning of iododiflunisal in the TTR channel is exclusively in the forward mode, this is, with the difluorophenyl ring occupying the inner part of the cavity and the salicylic ring the outer part. This is a common feature among other inhibitors having a biphenyl core molecule [62]. The same forward mode is also the single disposition that is seen in both **23b** and **22b** structures which show almost coincident spatial ring disposition (Figure 10 and Figure 11a). In both cases, the compounds are located further inside in the cavity than iododiflunisal. In sharp contrast, diflunisal is observed in the pocket sharing two orientations with equal probabilities, the one described as forward and a totally opposite where the rings swap positions that is called reverse mode.

**Figure 12:**
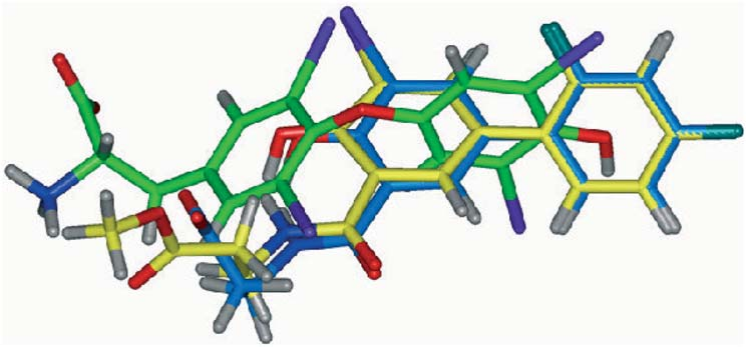
Superposition of TTR:iododiflunisal-betaAlaOH (22b) (blue) and TTR:iododiflunisal-betaAlaOMe (23b) (yellow) and TTR:T_4_ complexes (green). Iodine in iododiflunisal derivates is in the same region as I_3_ of T_4_.

**Figure 13:**
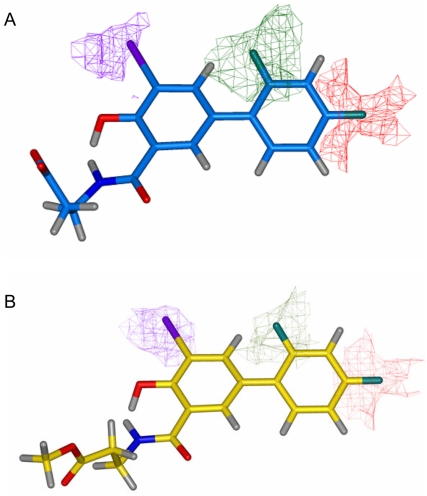
Affinity grids maps for TTR:iododiflunisal-betaAlaOH (22b) (A) and TTR:iododiflunisal-betaAlaOMe (23b) (B) complexes. Contour maps are drawn at −0.9 kcal/mol for fluorine and −6.3 kcal/mol for iodine. For clarity, only contour surrounding iodine and fluor substituents are shown and TTR structure is hidden. Iodine's contour is coloured in purple, fluorine countour coincident with HPB3 pocket is coloured in green, an the additional region located in the most inner part of the cavity along the two-fold symmetry axis is coloured in red.

The iodine atom in the iododiflunisal complex establishes close hydrophobic interactions with Leu17, Thr106, Ala108, Thr119 and Val121, thus, occupying the HBP1 pocket which is the outermost and more hydrophobic HBP. The innermost HBP pockets, HBP3 and HBP3′, in turn, closely interact with the fluorine atoms of the difluorophenyl ring. A further stabilizing interaction is found between the carbonyl group of Thr106 and iodine which closely resembles an halogen bond [63]. Similar but more optimized interactions than in the iododiflunisal complex are observed for the iodine atom in both crystal structures of **23b** and **22b** complexes. Thus, the iodine atom of these analogues interact with residues Leu17 and Ala 108 at distances ranging from 3.8 to 4.9 Å but it is more efficiently accommodated to the HBP1 because of a new hydrophobic interaction with Met13 and reinforcement of all the others. This fact is also in good agreement with GRID calculations. Interestingly, by superimposition of the conformations seen for **23b**, **22a** and **T_4_** in their crystal complexes, the position of the iodine atom of diflunisal analogues is identical to the iodine at C-3 in the thyroid hormone **T_4_** (Figure 12). This suggests that iodinated diflunisal analogues mimick some of the features of thyroid hormones. GRID also correctly predicted the interactions of the fluorine atoms. Thus, while fluorine at C-2′ is located in HBP3, the other, fluorine at C-4′, is placed in the most inner part of the binding cavity where GRID predicts a high energy binding site for fluorine (Figure 13).

Also important are the hydrophilic interactions detected in the iododiflunisal complex between the phenol and the carboxylic acid functions with the side chain of Lys15, a residue which is positioned at the entrance of the channel within close proximity to HBP1. This feature fixes the positions of the two Lys15 residues which in diflunisal are described as more disordered. These same additional hydrophilic interactions are also seen in **23b** and **22b** complexes but, here, are even more hardened owing to a new bifurcated hydrogen bond between the phenol group and both Lys15 and Lys15′ with distances in the range of 2.7 to 3.1 Å. A distinctive feature between **23b** and **22b** complexes comes from the location of the carboxyl group, thus, while the carboxylic acid of **22b** is asymmetrically positioned between Lys 15 (3.0 Å) and Lys 15′ (3.4 Å), the carbonyl of the ester of **23b** forms a water mediated hydrogen bond with Thr106. Moreover, additional hydrophobic interactions are build between the methyl group of this ester function and residues Pro24 and Ser52. All of them contribute to fix the ligand in the forward mode and to stabilize the tetramer explaining the superior binding affinity of compound **23b** over **22b** ([EC_50_ (**T_4_**)/EC_50_ (**23b**) = 3.4] *vs.* [EC_50_ (**T_4_**)/EC_50_ (**22b**) = 0.95] , see Figure 6 and Figure 7).

Other monomer-monomer interactions not reported in diflunisal but seen in iododiflunisal are a direct hydrogen bond between the two Ser117 residues. This motive is also evident in **23b** and **22b** where the binding of these analogues induce slight changes in the protein structure resulting in Ser117 reorientation as to form a strong hydrogen bond connecting the two monomers at a distance of 2.6 Å.

### Thyroid hormonal activity

To gain further insight on the therapeutic potential of these iodinated TTR fibrillogenesis inhibitors, *in vitro* binding tests of idodiflunisal to thyroid hormone receptors alfa and beta were carried out (data not shown). The almost negligible values of the binding constants suggest a possible lack of hormonal activity. This has been further confirmed by preclinical animal studies using a TTRV30M transgenic mice strain receiving 2.8 mg of iododiflunisal per day during 3 months. The animals did not show significant metabolic disfunctions. However, further preclinical tests are needed to validate these compounds as potential drugs for TTR related amyloidosis.

In conclusion, by mimicking the natural interactions between thyroid hormones and TTR and by using diflunisal as a model compound, the biochemical and biophysical data above discussed supports the hypothesis that iodine atoms inserted in TTR binding compounds is a crucial factor for the design of novel highly potent TTR fibrillogenesis inhibitors that one day become effective drugs for the treatment of TTR-related amyloidosis.

## Materials and Methods

*More detailed methods are included as* *Methods S1*.

### Computational GRID studies of TTR binding site and molecular modeling on TTR complexes

Using the crystallographic data reported for TTR in complex with T_4_, the geometry of the system was optimized using the all-atom force field [64] as implemented in AMBER6 [65]. The protein was embedded with a 3D grid and an halogen probe was placed in each grid point and the interaction energy between probe and protein was calculated obtaining a grid map by means of GRID software [66]. Map analysis with MINIM and FILMAP modules of GRID and visual inspection with grid module of Insight ll software [67] allowed to identify the minimal interaction energy points.

The complexes of TTR with iododiflunisal and compounds **23b** and **22b** have been analysed using similar computer protocols as in the affinity grid studies above in order to assess if halogens are located in favorable positions.

### Synthesis of TTR fibrillogenesis inhibitors

A library of 40 diflunisal analogues was prepared by individual synthesis. Compounds **1a** to **7b** were prepared by common organic chemistry reactions to modify the functional groups of diflunisal. The second group of diflunisal analogues (**8a** to **23b**) was obtained by conjugation of diflunisal with amino acids using standard peptide synthesis protocols in solution. The iodinated compounds of the “**b**” series were prepared from their non iodinated counterparts by reaction with IPy_2_BF_4_ [68]–[70].

### *In vitro* test of TTR fibrillogenesis inhibition

An *in vitro* turbidimetric test in a high troughput mode was used to assess the fibrillogenesis inhibition potency of the compounds of the library [54]. The mutant protein TTRY78F was incubated with various concentrations of test compounds in 96 well microplates. After lowering the pH of the solutions to 4.2, the incubation was extended for 1.5 h while recording the absorbance at 340 nm at 1 min intervals. Plots of initial rates of fibril formation versus test compounds concentrations followed exponential equations from which RA (%) and IC50 parameters were derived [54] (Figure S1).

### Binding competition between diflunisal analogues and T_4_

To evaluate the relative binding affinity of the compounds for TTR an already described procedure was followed [55]. Wild type recombinant TTR was incubated with trace amounts of ^125^I-T_4_ in the presence of increasing amounts of test compounds. Protein bound ^125^I-T_4_ was separated from the media by gel filtration and measured by scintillation. Competition curves allowed to calculate the relative T_4_ displacement potencies defined as EC_50_ of **T_4_**/EC_50_ of test compound for each inhibitor (Figure S2).

### Binding selectivity for plasma proteins

Human plasma was incubated with labeled T_4_ (^125^I-T_4_) in the presence of each inhibitor. After separation of plasma proteins by native polyacrylamide gel electrophoresis (PAGE), qualitative T_4_ displacement binding from TBG, ALB and TTR (main plasma T_4_ binding proteins) was measured by phosphorimaging of the dried gel [55] (Figure S3).

### Monitoring tetramer dissociation by circular dichroism

It is known that the dissociation of the tetrameric form of TTRwt takes an average of 42 h and the resulting monomers quickly unfold 500.000 times faster [71]. Therefore the rate of dissociation can be estimated by lining it to unfolding which is irreversible in 6M urea. Thus, owing that the tetramer dissociation is the rate limiting step for fibrilogenesis and assuming the kinetic stabilizing mechanism of inhibition, good inhibitors should slow tetramer dissociation.

Although slow tetramer dissociation is not detectable by far-UV CD spectroscopy, this technique is very much suitable at monitoring the much faster unfolding step.The CD spectra of TTR corresponds to a typical β-sheet reach protein with a Cotton effect centered at 210 nm and a shoulder at 217 nm. This band gradually looses intensity as protein denaturation advances, therefore meaningful changes in the β-sheet content of the protein are normally recorded in the region of 210–250 nm. Below this region (190–210 nm) other interesting structural changes are masked by the high concentrations of urea required for denaturation. Results are here presented in unfolded fraction units which are calculated from each spectra assuming than at time zero the elipticity recorded corresponds to the fully folded protein while at 200 h this parameter agrees with a totally unfolded state.

Accordingly, the stabilizing effect of the tetrameric form of both TTRwt and TTRY78F by iodine atoms was evaluated on urea-induced dissociation experiments monitoring the secondary structural changes using far-UV circular dichroism techniques by assessing the performance of iododiflunisal (IDIF, **1b**) versus diflunisal (DIF, **1a**). Both proteins were incubated in buffered solutions of 6M urea in the presence and absence of 1:2 ratios of protein to inhibitor and far-UV CD spectra (210–220 nm) were intermitently recorded for a period of 200 hours. Note that because TTR is a heat-resistant protein incubation temperature was set at 25°C rather than the usual 37°C.

### Protein complex preparation and crystallization

TTR protein solutions were incubated for 24 h with 10 fold -molar excess of the inhibitors. Crystals of the complexes were obtained by hanging-drop vapor diffusion techniques. Crystals for data collection were transferred to glycerol solutions and flash frozen into liquid nitrogen.

### Data collection, processing and refinement

Difracction data was collected at the European Synchroton Radiation Facility (Grenoble, France). Crystal orientation and integration of reflexions were performed with MOSFLM [72] and scaling and merging of reflections with SCALA and TRUNCATE [73]. The structures were determined with Phaser [74] using the coordinates of TTRT119M [75]. Refinement was conducted by means of CNS [76] and Turbo FRODO [77] programs. Atomic coordinates of binding compounds were obtained from the HIC UP database [78] and the model further refined with REFMAC [79] using the CCP4i program suite [73]. The quality of final model was checked using PROCHECK [80].

## Supporting Information

Figure S1 Kinetic turbidity assay with Y78F-hTTR. Relative initial rates of fibril formation (v_0_ , %) are plotted against inhibitor concentration for selected compounds (1a,1b, 17a, 17b, 20a, 20b, 22a, 22b, 23a, 23b).
(1.24 MB TIF)

Figure S2 Plots of the ratio T_4_ bound to TTR/total T_4_ against the logarithm of inhibitor concentration for selected compounds (1a,1b, 17a, 17b, 20a, 20b, 22a, 22b, 23a, 23b).
(5.57 MB TIF)

Figure S3 Polyacrylamide gel electrophoresis of plasma proteins. The plasma was incubated with ^125^I-T_4_ in the presence of the indicated compounds. C: plasma incubated with ^125^I-T_4_ with no competitor. The migration of the main T_4_ binding plasma proteins, TBG, Albumin, and TTR is indicated.
(2.55 MB TIF)

Text S1 Data collection and refinement statistics
(0.21 MB DOC)

Methods S1 Iodinated TTR inhibitors
(1.00 MB DOC)
