# Supplementary figures and images for: Iodine Atoms: A New Molecular Feature for the Design of Potent Transthyretin Fibrillogenesis Inhibitors
Source: PLoS One. 2009 Jan 6;4(1):e4124. doi: 10.1371/journal.pone.0004124 (PMC2607018; doi:10.1371/journal.pone.0004124)

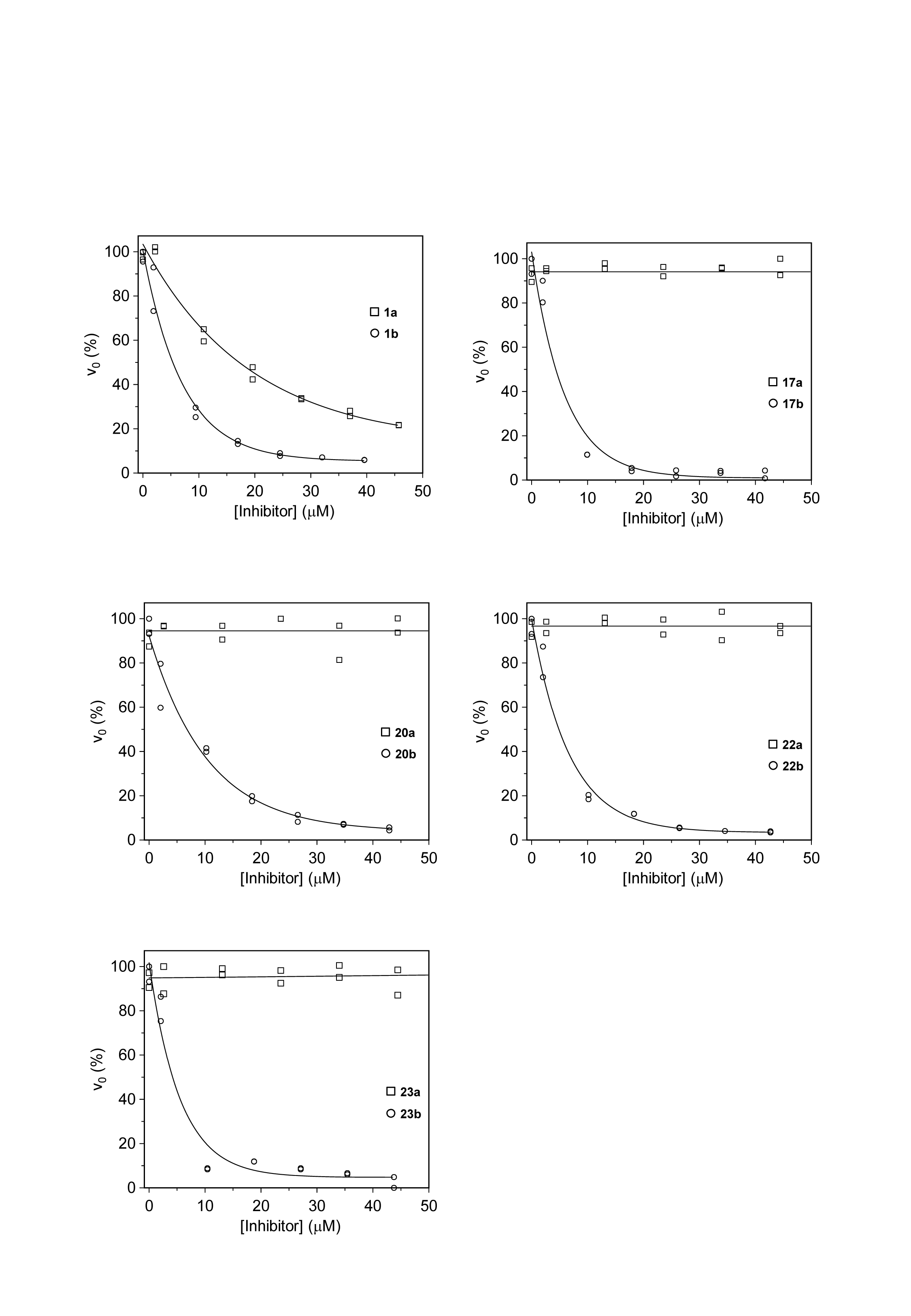

Supplement: Figure S1 — Kinetic turbidity assay with Y78F-hTTR. Relative initial rates of fibril formation (v0 , %) are plotted against inhibitor concentration for selected compounds (1a,1b, 17a, 17b, 20a, 20b, 22a, 22b, 23a, 23b). (1.24 MB TIF) [file pone.0004124.s001.tif]

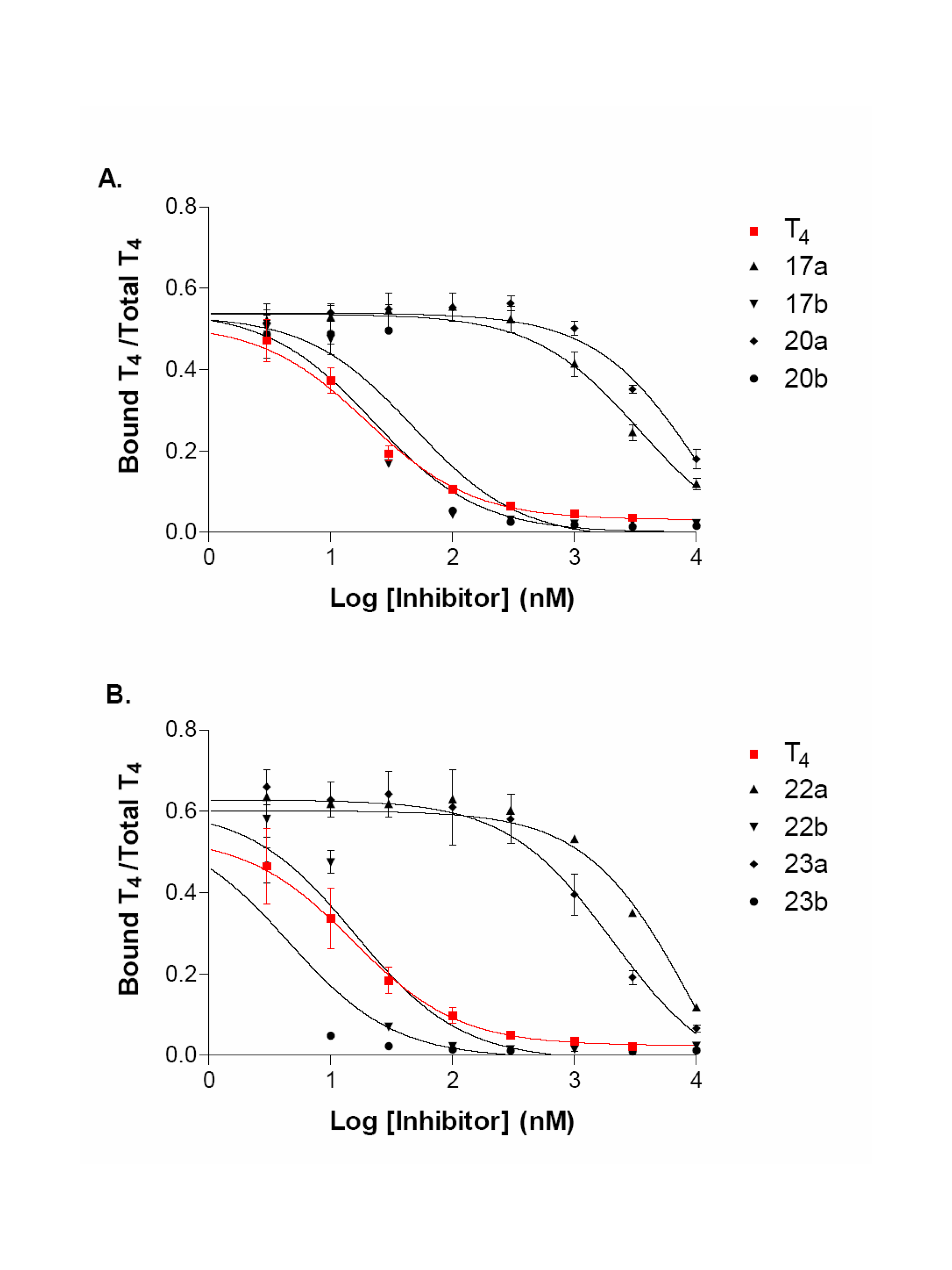

Supplement: Figure S2 — Plots of the ratio T4 bound to TTR/total T4 against the logarithm of inhibitor concentration for selected compounds (1a,1b, 17a, 17b, 20a, 20b, 22a, 22b, 23a, 23b). (5.57 MB TIF) [file pone.0004124.s002.tif]

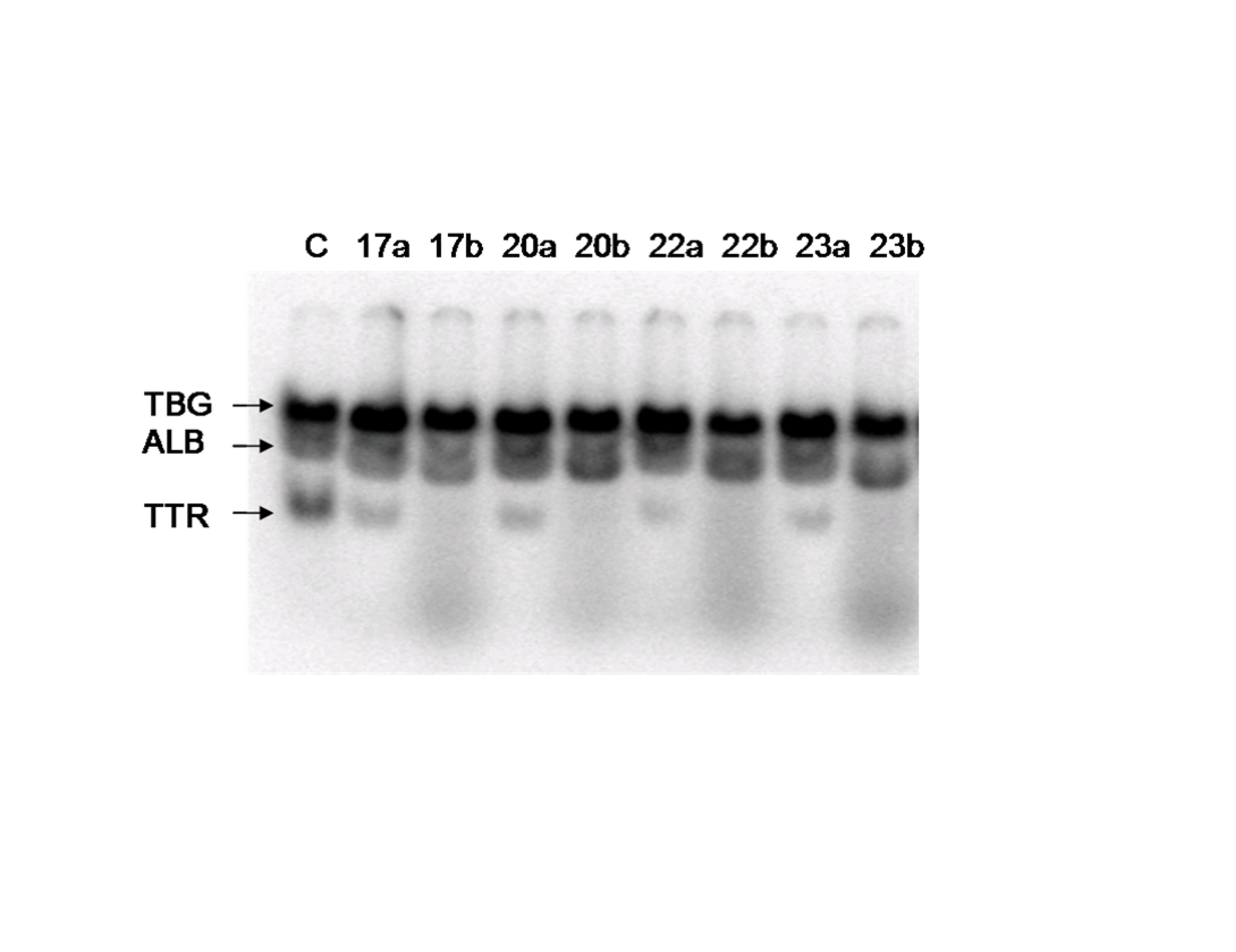

Supplement: Figure S3 — Polyacrylamide gel electrophoresis of plasma proteins. The plasma was incubated with 125I-T4 in the presence of the indicated compounds. C: plasma incubated with 125I-T4 with no competitor. The migration of the main T4 binding plasma proteins, TBG, Albumin, and TTR is indicated. (2.55 MB TIF) [file pone.0004124.s003.tif]
