# Supplementary material for: Iodine Atoms: A New Molecular Feature for the Design of Potent Transthyretin Fibrillogenesis Inhibitors
Source: PLoS One. 2009 Jan 6;4(1):e4124. doi: 10.1371/journal.pone.0004124 (PMC2607018; doi:10.1371/journal.pone.0004124)
Supplement: Text S1 — Data collection and refinement statistics (0.21 MB DOC) [file pone.0004124.s004.doc]

|  | TTR:iododiflunisal-betaAlaOMe (**23b**) | TTR:iododiflunisal-betaAlaOH (**22b**) |
| --- | --- | --- |
| **Data collection** |  |  |
| Space Group | P2_1_2_1_2 | P2_1_2_1_2 |
| Unit Cell dimensions (Å) | a=42.2 b=85.1 c=63.1 | a=43.0 b=85.7 c=63.6 |
| Resolution range (Å) | 50.97-1.85 | 63.76-1.80 |
| No. of observations (total/unique) | 189703/ 20049 | 162528 / 22496 |
| Multiplicity (overall/last shell) | 9.5 / 6.8 | 7.2 / 7.4 |
| Rmerge (overall/last shell) | 5.6 / 23.4 | 5.8 / 16.0 |
| Completeness (%)(overall/last shell) | 99.8 / 99.2 | 99.9 / 98.7 |
| I/σ(I) (overall/last shell) | 32.4 / 8.2 | 27.0 / 9.9 |
| Mathews Coefficient (Å^3^Da^-1^) | 2.10 | 2.11 |
| Solvent content (%) | 41.0 | 41.1 |
| **Structure refinement** |  |  |
| Rfactor / Rfree | 19.9 / 21.5 | 19.9 / 23.0 |
| No. of unique reflections  (working / test set) | 18982 / 950 | 22499 / 1136 |
| Water molecules | 132 | 157 |
| Residues with alternate conformations | S115A | S85A, N27B |
| Total number of atoms | 1940 | 1987 |
| Average protein B-factor (Å^2^) | 15.3 | 16.7 |
| Average main-chain B-factor (Å^2^) | 13.8 | 15.2 |
| Average side-chain B-factor (Å^2^) | 17.1 | 18.2 |
| Average ligand B-factor (Å^2^) | 16.1 | 24.3 |
| R.m.s. bonded B’s (Å^2^) | 16.5 | 16.8 |
| R.m.s. deviations from ideal values  Bonds (Å)  Angles (º) | 0.017  1.3 | 0.018  1.4 |
| Ramachandran plot statistics  Most favoured regions (%)  Additionally allowed regions (%) | 92.5  7.5 | 91.0  9.0 |
