# Supplementary material for: Iodine Atoms: A New Molecular Feature for the Design of Potent Transthyretin Fibrillogenesis Inhibitors
Source: PLoS One. 2009 Jan 6;4(1):e4124. doi: 10.1371/journal.pone.0004124 (PMC2607018; doi:10.1371/journal.pone.0004124)
Supplement: Methods S1 — Iodinated TTR inhibitors (1.00 MB DOC) [file pone.0004124.s005.doc]

**SUPPORTING INFORMATION**

**Iodine Atoms: a New Molecular Feature for the Design of Potent Transthyretin Fibrillogenesis Inhib­itors.**

Teresa Mairal1[[1]](#footnote-2)+, Joan Nieto2[[2]](#footnote-3)+, Marta Pinto^3^, Maria Rosário Almeida^4^, Luis Gales^4^, Alfredo Ballesteros^5^, José Barluenga^5^, Juan J. Pérez^3^, Jesús T. Vázquez^6^, Nuria B. Centeno^7^, Maria Joao Saraiva^4^, Ana M. Damas^4^, Antoni Planas^2^, Gemma Arsequell^1^, Gregorio Valencia1[[3]](#footnote-4).

^1^Unit of Glycoconjugate Chemistry, Institut de Química Avançada de Catalunya, I.Q.A.C.-C.S.I.C., Jordi Girona 18-26, 08034 Barcelona (Spain); and ^2^Laboratory of Biochemistry, Institut Químic de Sarrià, Universitat Ramon Llull, Via Augusta, 390, 08017 Barcelona (Spain); and ^3^Departamento de Ingenieria Química, ETSEIB-Universitat Politècnica de Barcelona, Avda. Diagonal, 647, 08028 Barcelona (Spain); and ^4^IBMC - Instituto de Biologia Molecular e Celular, Universidade do Porto, Rua do Campo Alegre 823, 4150-180 and ICBAS - Instituto de Ciências Biomédicas Abel Salazar, Universidade do Porto, Largo Prof. Abel Salazar, 2, 4099-003 Porto (Portugal); and ^5^Instituto Universitario de Química Organometálica “Enrique Moles”, Unidad Asociada al C.S.I.C. Universidad de Oviedo, Julián Clavería, 8; 33006 Oviedo (Spain); and ^6^Instituto Universitario de Bio-Orgánica “Antonio González”, Universidad de La Laguna, Avda. Astrofísico Fco. Sánchez, 2, 38206 La Laguna, Tenerife (Spain); and ^7^Computer-Assisted Drug Design Laboratory, Research Group on Biomedical Informatics (GRIB) IMIM-Universitat Pompeu Fabra, Dr. Aiguader 88, 08003 Barcelona (Spain).

**Keywords**: transthyretin, iodination, amyloid, diflunisal analogs, NSAID

***Synthesis of analogs.***

Amides **2a** and **2b** were obtained by ammonolysis of the corresponding carboxylic acids **1a** and **1b**, respectively. Methyl esters (**3a** and **3b**) and ethyl esters (**4a** and **4b**) were obtained from the corresponding carboxylic acids **1a** and **1b** by using thionyl chloride with either methanol or ethanol. Phenol acetates **7a** and **7b** were prepared by acetylation of **1a** (diflunisal) and **1b** (iododiflunisal), respectively, using anhydride acetic in pyridine. The biphenyl aniline derivative **5a** was prepared by aqueous Suzuki cross-coupling reaction between 2,4-difluorophenylboronic acid and 5-iodoanthranilic acid using a 10% palladium acetate as catalyst and a 2M solution of sodium carbonate. The corresponding methyl esters **6a** and **6b** were prepared from the carboxylic acid derivatives **5a** and **5b**, respectively, using thionyl chloride in methanol. All the corresponding iodinated derivatives were prepared using the iodinating reagent IPy_2_BF_4_ in CH_2_Cl_2_ (Barluenga’s reagent) ([[4]](#endnote-2)). Diflunisal amino acid conjugates (from **8a** to **21a**) were obtained by coupling reaction between diflunisal and alpha-amino acid derivatives (either free or conveniently protected). The coupling reagent used was *N,N’*-dicyclohexyl carbodiimide (DCC) in the presence of 1-hydroxybenzotriazol (HOBt) for the amide bond formation. The corresponding iodinated derivatives (**8b** to **21b**) were obtained from the corresponding conjugated derivatives (**8a** to **21a**) by iodination using the above mentioned reaction with Barluenga’s reagent. Conjugation of diflunisal with beta-alanine (free or as methyl ester) was done using the same coupling procedure with DCC and HOBt in CH_2_Cl_2_. The coupling reaction yielded derivatives **22a** (from coupling to free beta-alanine) and **23a** (by coupling to the methyl ester of beta-alanine), which were iodinated with Barluenga’s reagent to yield **22b** and **23b**, respectively.

**SYNTHESIS OF ANALOGUES.**

HPLC gradient GEN1 is specified in the following table. The solvents used are A: 0.1% TFA in H_2_O and B: 0.1% TFA in CH_3_CN. From 50% of A and 50% of B to 10% of A and 90% of B in 25 min, then to 50% of A and 50% of B in 2 min.

**1. TYPICAL IODINATION PROCEDURE:**

**Example: Synthesis of 2',4'‑difluoro‑4‑hydroxy‑5‑iodo‑[1,1']‑biphenyl‑3‑carboxylic acid (Iododiflunisal, 1b).**

To a solution of 200 mg (0.80 mmol) of diflunisal in 5 ml of dichloromethane, 357 mg (1.2 mmol) of IPy_2_BF_4_ were added to obtain a substrate/reagent ratio of 1:1.5 equivalents. The reac­tion was left under stirring at room temperature and monitored by HPLC until the starting material was fully converted into the iodo derivative. This occurs in a typical time of 1.5 h. After diluting with dicloromethane the reaction mixture was successively washed with HCl 1N and sodium thiosulfate 0.1N solu­tion. Drying over MgSO_4_ and evaporation of the organic layer yielded a residue of a 98% typical purity which was further purified by column chromatography on silica gel using HCCl_3_/MeOH gradient mixture or HPLC.

2. TYPICAL AMIDATION (CARBOXAMIDE PREPARATION) PROCEDURE:

**Example: Synthesis of 2',4'‑difluoro‑4‑hydroxy‑[1,1']‑biphenyl‑3‑carboxamide (2a).**

A solution of 1 g (3.9 mmol) of diflunisal and 0.73 g (3.9 mmol) of pentafluorophenol in 20 ml of acetonitrile was prepared and 10 ml of *N,N'*‑diisopropylcarbodiimide were added. The reaction was kept at 0ºC under stirring for 1 hour and 0.63 g (8.3 mmol) of ammoni­um hydrogencarbonate in the minimum volume of water added. The reaction mixture was stirred until the pentafluorophenol ester was completely converted into the amide. The reaction was futher diluted with water and extracted wiht dichloromethane. The organ­ic layer was dried and evaporated to dryness and the residue purified by column chromatography on silica gel using a HCCl_3_/MeOH gradient mixture of 40:1 up to 10:1 (v/v).

3: TYPICAL ESTERIFICATION PROCEDURE:

**Example: Synthesis of 2',4'‑difluoro‑4‑hydroxy‑[1,1']‑biphenyl‑3‑carboxylic methyl ester (3a).**

Under nitrogen atmosphere, a dropwise addition of 2.5 ml (34.3 mmol) of thionyl chloride to 10 ml of cool (‑10ºC) and stirred MeOH (or in ethyl esters EtOH) was effected. The mixture was further stirred for 15 min and 1 g (3.99 mmol) of solid diflunisal was added at once. The reaction mixture was refluxed for 24 h and later the solvent evaporated to dryness. Removal of volatile byproducts from the reaction mixture was performed by successive addition‑evaporation aliquots of methanol. The crude material was purified by crystallization and recrystallization from acetone/water mixtures.

4: TYPICAL ACETYLATION PROCEDURE:

**Example: Synthesis of 4‑acetoxy‑2',4'‑difluoro‑[1,1']‑biphenyl‑3‑carboxylic acid (7a).**

To a cooled (0ºC) and stirred solution of 5 ml of pyridine and 1 ml of acetic anhydride, 200 mg (0.79 mmol) of solid diflunisal were added. The reaction was left to proceed at room temperature during 24 h. After removal of the solvent, the residue was sus­pended in methylene chloride and washed with 3N aqueous HCl. The organic layer was dried and the solvent evaporated. The resulting crude material was purified by column chromatography on silica gel using HCCl_3_/MeOH as elution solvent.

5: TYPICAL COUPLING METHOD FOR THE PREPARATION OF DIFLUNISAL‑ (ALFA AND BETA) AMINO ACID CONJUGATES:

**Example: Synthesis of *N*‑(2',4'‑difluoro‑4‑hydroxy‑[1,1']‑biphe­nyl‑3‑carbonyl]-glycine *tert*‑butyl ester (8a).**

To a stirred solution of 300 mg (1.19 mmol) of diflunisal in 10 ml of dichloromethane, a second solution of 285 mg (2.07 mmol) of hydroxybenzotriazole in a minimum quantity of dichoromethane was added. Further addition of 199 mg (1.19 mmol) of glycine *tert*-butyl ester hydrochloride in 10 ml of dichloromethane containing 338 l (1.97 mmol) of diisopropylehtylamine was next effected. The coupling reaction was started by addition of 295 mg (1.43 mmol) of *N,N’*-dicyclohehylcarbodiimide disolved in 10 ml of dichloromethane. The reaction was stirred at room temperature and monitored by TLC and HPLC. Diflunisal was fully converted on the conjugate after 3 hours of reaction. The insoluble urea was filtered off and the organic layers was evaporated to dryness. The residue was purified by column chromatography on silica gel using hexane/diethyl eter (3:1) as mobile phase.

6: TYPICAL PROCEDURE FOR THE REMOVAL OF *TERT*‑BUTYL ESTER GROUPS ON THE CONJUGATES.

**Example: Synthesis of *N*-[2',4'-difluoro-4-hydroxy-[1,1']-biphenyl-3-carbonyl]-glycine.**

The *tert*-butyl ester hydrolisis of the precursor product of the title compound was carried out on a 200 mg sample using a 15 ml mixture of trifluoroacetic acid/dichloromethane (1:1) mixture. TLC monitoring indicates that the reaction is complete after 1.5 hours at room temperature.

7: TYPICAL PROCEDURE FOR THE REMOVAL OF ETHYL OR METHYL ESTER GROUPS ON THE CONJUGATES

**Example: Synthesis of *N*-[2',4'-difluoro-4-hydroxy-[1,1']-biphenyl-3-carbonyl]-L-alanine.**

The title compound was prepared from its methyl ester precursor by saponification. A solution of 0.1 M of the ester was prepared using a tertiary solvent mixtures of tetrahydrofurane/methanol/water (3:1:1) that was cooled to 0ºC and a LiOH (4 equiv) in water was next added. The rection was stirred at room temperature and monitored by TLC. After 4 hours the reaction was complete and then 1N HCl was added until pH = 4. Organic solvent evaporation yielded an aqueous phase that was extracted with ethyl acetate which was dried over Na_2_SO_4_ and the solvent was evaporated off. The residue was finally purified by column chromatography on silica gel using a chloroform/methanol (40:1) mixture.

**2’,4’-difluoro-4-hydroxy-[1,1’]-biphenyl-3-carboxylic acid (diflunisal) (1a)**

**Formula:** C13H8F2O3

**HPLC (GEN1) RT:** 8.40 min.

**^1^H-RMN (500 MHz; DMSO-d6) δ (ppm):** 7.92-7.91 (m, 1H), 7.66-7.63 (m, 1H), 7.54 (dt, *J*=6.5, 9.0 Hz, 1H), 7.29-7.25 (m, 1H), 7.16-7.12 (m, 1H), 7.05 (d, *J*=8.5 Hz, 1H).

**^13^C-RMN (125.7 MHz; DMSO-d6) δ (ppm):** 171.4, 161.4 (dd, *J_C_*_F_=12.3, 247.0 Hz), 160.6, 158.9 (dd, *J_CF_*=12.3, 248.3), 135.6 (d, *J_CF_*=2.8 Hz), 131.4 (dd, *J_CF_*=4.5, 9.5 Hz), 130.2 (d, *J_CF_*=2.9 Hz), 125.1, 123.7 (dd, *J_CF_*=3.9, 13.6 Hz), 117.5, 113.2, 111.9 (dd, *J_CF_*=3.3, 21.2 Hz), 104.3 (t, *J_CF_*=26.6 Hz).

**MS (ESI^-^) m/z** 249 (M-H)^-^

**2’,4’-difluoro-4-hydroxy-5-iodo-[1,1’]-biphenyl-3-carboxylic acid (iododiflunisal) (1b)**

**Formula:** C13H7F2IO3

**HPLC (GEN1) RT:** 13.83 min.

**^1^H-NMR (500 MHz; DMSO-d6) δ (ppm):** 8.10-8.09 (m, 1H), 7.93-7.92 (m, 1H), 7.57 (dt, *J*=6.5, 9.0 Hz, 1H), 7.31-7.27 (m, 1H), 7.16-7.12 (m, 1H).

**^13^C-NMR (125.7 MHz; DMSO-d6) δ (ppm):** 171.2, 161.6 (dd, *J_CF_*=12.6, 247.6 Hz), 159.7, 158.9 (dd, *J_CF_*=12.3, 248.5 Hz), 144.1, 131.5 (dd, *J_CF_*=4.5, 9.5 Hz), 130.4, 126.8, 122.4 (dd, *J_CF_*=3.6, 13.3 Hz), 113.2, 111.9 (d, *J_CF_*=20.9 Hz), 104.3 (t, *J_CF_*=26.4 Hz), 85.9 (C-I).

**ESI-MS (ESI^-^) m/z** 375 (M-H)^-^

**2’,4’-difluoro-4-hydroxy-[1,1’]-biphenyl-3-carboxamide (2a)**

**Formula:** C13 H9 NO2 F2

**HPLC (GEN1)** RT: 6.62 min

**^1^H-NMR** (500 MHz, acetone-d^6^): δ (pp m): 8.33 (s, 1H, NH), 8.03 (s,1H), 7.61 (d, *J* = 8.5 Hz,1H), 7.65 (s, 1H, NH), 7.56 (dd, *J* = 8.5, 15.5 Hz,1H), 7.13-7.08 (m, 2H), 7.0 (d, *J* =8.5 Hz, 1H)

**^13^C-NMR**  (125.7 MHz, acetone-d^6^): δ (ppm): 173.7, 162.9 (dd,*J*_CF_=11.8, 259.1 Hz), 162.8, 160.5 (dd, *J*_CF_=11.6, 248.4 Hz), 135.6 (d, *J*_CF_= 3.4 Hz), 132.6 (dd, *J*_CF_=4.5, 9.5 Hz), 128.8, 125.9,125.4 (d, *J*_CF_= 9.8 Hz), 118.8, 114.9, 112.5 (dd, *J*_CF_= 3.6, 20.9 Hz), 104.9 (t, *J*_CF_= 25.89 Hz)

**IR (cm^-1^):** ν_max_. 3497(ν_OH_), 3337 (ν_NH2_), 3194 (ν_NH2_), 1490 (ν_C=O_)

**ESI-MS:** M^+^: 249.06; m/z=249.06 (M^+^ ,100%)

**2’,4’-difluoro-4-hydroxy-5-iodo-[1,1’]-biphenyl-3-carboxamide (2b)**

**Formula:** C_13_ H_8_ NO_2_ F_2_I

**HPLC (GEN1)** RT: 12.08 min.

**^1^H- NMR** (500 MHz, acetone-d^6^): δ(ppm): 8.11 (s,1H), 8.03 (s, 1H), 7.59 (dd, *J* = 8.5, 15.5 Hz,1H), 7.18-7.11 (m, 2H)

**13C- NMR**  (125.7 MHz, acetone-d^6^ ): δ (ppm): 171.8, 163.2 (dd,*J*_CF_=12.2, 247.63 Hz), 161.9, 160.4 (dd,*J*_CF_=12.44, 247.25 Hz), 144.6 (d, *J*_CF_= 3.4 Hz), 132.7 (dd, *J*_CF_=4.52, 9.55 Hz), 129.1, 127.7, 114.7, 112.6 (dd, *J*_CF_= 3.93, 21.24 Hz), 104.2 (t, *J*_CF_= 26.02 Hz), 86.7(C-I).

**IR (cm^-1^):** ν_max._3189 (ν_C=O_), 1672 (amide, ν_C=O_), 1438 (amide, ν_C=N_)

**MALDI-TOF-MS:** M^+^: 374.96 (α-cyano-4-hydroxycinnamic acid as matrix) m/z=374.96 (M^+^, 100%)

**2’,4’-difluoro-4-hydroxy-[1,1’]-biphenyl-3-carboxylic methyl ester (3a)**

**Formula:** C_14_ H_10_ NO_3_ F_2_

**HPLC (GEN1)** RT: 17.88 min.

**^1^H-NMR** (500 MHz,CDCl_3_): δ(ppm): 10.83 ( s,1H), 7.97 (dt, *J*: 2.5, 1.5 Hz, 1H) 7.60 (dt, *J*=8.5,2.0 Hz,1H), 7.36 (dd, 1H, *J*=8.5, 15 Hz,1H) 7.06 (d, *J* = 8.5 Hz,1H), 6.92 (m, 2H), 3.97 (s, CH_3_)

**^13^C-NMR**  (125.7 MHz, CDCl_3_): δ (ppm): 170.4,162.2 (dd, *J*_CF_=11.7, 249.3 Hz), 161.2, 159.7 (dd, *J*_CF_=11.7, 249.3 Hz), 136.2 (d,*J*_CF_=2.9 Hz),131.1 (dd, *J*_CF_=4.7, 9.55 Hz), 130.2 (d,*J*_CF_=2.8 Hz), 126.1, 124.18, 117.7, 113.2, 111.6 (d, *J*_CF_= 3.89, 21.1 Hz), 104.2 (t, *J*= 25.4Hz), 52.3 (CH_3_).

**MALDI-TOF-MS:** MH^+^: 265.06 (α-cyano-4-hydroxycinnamic acid) m/z= 264.06 (M^+^,100%)

**2’,4’-difluoro-4-hydroxy-5-iodo-[1,1’]-biphenyl-3-carboxylic methyl ester (3b)**

### Formula: C_14_ H_9_ O_3_ F_2_I

**HPLC** (GEN1) RT: 20.27 min.

**^1^H- NMR** ( 500 MHz, CDCl_3_): δ ( ppm): 11.69 ( s,1H), 8.09 (t, *J*=2 Hz,1H), 7.98 (t, *J* = 2 Hz ,1H), 7.33 (dd, J=8.5, 14.5 Hz,1H), 7.26-6.92 (m, 2H), 3.97 (s, CH_3_)

**^13^C- NMR**  (125.7 MHz, CDCl_3_): δ en ppm: 170.1, 162.6 (dd, *J*_CF_=11.7, 249.8 Hz), 159.9, 159.7 (dd, *J*_CF_=11.9, 250.4 Hz), 145.3, 131.2 (dd, *J*_CF_=4.7,9.4 Hz), 130.6 (d, *J*_CF_=3.1 Hz), 127.9, 122.9

(dd, *J*_CF_=3.9, 13.7 Hz), 112.5,111.9 (dd, *J*_CF_=3.6, 20.9 Hz), 104.6 (t, *J*=26.12 Hz), 85.59, 53.1(CH_3_).

**MALDI-TOF-MS:** MH^+^: 390.76 (matrix acid α-cyano-4-hydroxycinnamic acid) m/z: 389.96( M^+^,100%)

**2’,4’-difluoro-4-hydroxy-[1,1’]-biphenyl-3-carboxylic acid ethyl ester (4a)**

### Formula: C_15_ H_12_ O_3_ F_2_

**HPLC** (GEN1) RT: 20.45 min.

**^1^H-NMR** ( 400 MHz, CDCl_3_): δ (ppm): 10.94 (s,1H), 7.97 (s,1H), 7.59 (d, *J*=10.4 Hz.,1H), 7.35 (s,1H), 7.03 (d,*J*=8.5 Hz,1H), 6.97-6.88 (m, 2H), 4.43 (dd, *J*= 14.4, 7.2 Hz, 2H, CH_2_), 1.42 (t,*J*=7.2 Hz,3 CH_3_).

**^13^C-NMR**  (100.56 MHz, CDCl_3_): δ (ppm): 170.2, 163.5 (dd, *J*_CF_ =11.2, 249.1 Hz) ,161.1, 160.1 (dd, *J*_CF_ =11.2, 249.1 Hz), 136.2 (d, *J*_CF_=2.5 Hz), 131.2 (dd, *J*_CF_ =4.6, 9.3 Hz), 130.3 (d, *J*_CF_ = 2.6 Hz), 128.9, 124.3 (t, *J*_CF_= 9.8 Hz), 117.9, 112.8, 111.7 (dd, *J*_CF_ =3.8,20.9 Hz.), 104.6 (t,*J*_CF_= 26.42 Hz), 61.8,14.3 .

**ESI-MS:** M^+^: 278.1 (M^+^: 278.1)

**2,4’-difluoro-4-hydroxy-5-iodo-[1,1’]-biphenyl-3-carboxylic acid ethyl ester (4b)**

### Formula: C_15_ H_11_ O_3_ F_2_I

**HPLC** (GEN1) RT: 25.28 min.

**^1^H- NMR** ( 500 MHz, CDCl_3_ ): δ (ppm): 11.8 (s,1H), 8.07 (s, 1H), 7.98 (s, 1H), 7.34 (dd, *J*= 9.0, 15 Hz, 1H), 6.9-6.8 (m,2H), 4.4 (d, *J*=7.0,14.0 Hz,2H), 1.42 (t, *J*=7.0 Hz,CH_3_ )

**^13^C-NMR**  (100.56 MHz, CDCl_3_): δ (ppm): 169.6, 162.5 (dd, *J*_CF_ =11.5, 249.5 Hz), 160.1, 159.6 (dd, *J*_CF_ =11.4, 250.2 Hz), 145.2 (d, *J*_CF_ =2.3 Hz), 131.2 (dd, *J*_CF_ =3.8, 9.1 Hz.), 130.5 (d, *J*_CF_ =2.2 Hz), 127.8, 123.1 (t, *J*_CF_ = 3.1 Hz), 112.7, 111.9 (dd, *J*_CF_ =3.0, 20.6 Hz), 104.68(t, *J*_CF_ =26.1 Hz),85.6 (C-I), 62.4 , 14.3

**ESI-MS:** MH^+^: 405.0 m/z= 403.97 (M^+^, 100%)

**2’,4’-difluoro-4-amino-[1,1’]-biphenyl-3-carboxylic acid (5a)**

**Formula:** C13H9F2NO2

**HPLC (GEN1) RT:** 6.76 min.

**^1^H-NMR (500 MHz; CD_3_COCD_3_) δ (ppm):** 8.04 (m, 1H), 7.51 (dt, *J*=6.5, 8.5 Hz, 1H), 7.47 (dt, *J*=2.0, 8.5 Hz, 1H), 7.10-7.04 (m, 2H), 6.93-6.91 (m, 1H).

**^13^C-NMR (125.7 MHz; CD_3_COCD_3_) δ (ppm):** 169.9, 162.5 (dd, *J_CF_*= 11.9, 246.0 Hz), 160.4 (dd, *J_CF_*=12.1, 247.9 Hz), 152.3, 135.3 (d, *J_CF_*=2.9 Hz), 132.6 (d, *J_CF_*=3.3 Hz), 131.9 (dd, *J_CF_*= 5.0, 9.7 Hz), 125.9 (dd, *J_CF_*= 3.6, 13.6 Hz), 122.3, 117.6, 112.4 (dd, *J_CF_*=3.6, 21.1 Hz), 110.4, 104.9 (t, *J_CF_*=26.4 Hz).

**ESI-MS (ESI^-^) m/z** 248.1 (M-H)^-^

**2’,4’-difluoro-4-amino-3-iodo-[1,1’]-biphenyl-3-carboxylic acid (5b)**

**Formula:** C13H8F2INO2

**HPLC (GEN-1) RT:** 12.96 min.

**^1^H-NMR (500 MHz; CD_3_OD) δ (ppm):** 8.06 (m, 1H), 7.98 (m, 1H), 7.46-7.41 (m, 1H), 7.04-6.99 (m, 2H).

**^13^C-NMR (125.7 MHz; DMSO-d6) δ (ppm):** 168.5, 161.2 (dd, *J_CF_*=12.4, 246.6 Hz), 158.8 (dd, *J_CF_*=12.2, 247.7 Hz), 149.6, 143.3, 131.8 (d, *J_CF_*=1.9 Hz), 131.1 (dd, *J_CF_*=5.4, 15.9 Hz), 122.9 (dd, *J_CF_*=5.5, 13.8 Hz), 122.5, 111.8 (d, *J_CF_*=20.6 Hz), 110.7, 104.3 (t, *J_CF_*=26.9 Hz), 86.1 (C-I).

**ESI-MS (ESI^-^) m/z** 374.1 (M-H)^-^

**2’,4’-difluoro-4-amino-[1,1’]-biphenyl-3-carboxylic acid methyl ester (6a)**

Suzuki reaction: To a suspensión of 0.102 g (0.12 mmol) of PdCl_2_(dppf) in dioxane, 0.214 g (1.36 mmol) of 2,4-difluorophenyl boronic acid, 1.3 mL of 2M aqueous solution of Na_2_CO_3_ (2.57 mmol) and 2-amino-5-bromobenzoic acid methyl ester (0.300 g, 1.30 mmol) are sequentially added. The mixture is heated at 100ºC and the reaction monitored by HPLC. The solvent is evaporated, filtered through Celite^®^ and the crude reaction purified by column chromatography in silica gel using and Hexane/AcOEt (6:1) mixture yielding 0.167 g (49% yield) of pure product.

**Formula:** C_14_H_11_F_2_NO_2_

**HPLC (GEN1) RT:** 13.65 min.

**^1^H-NMR (500 MHz; CDCl_3_) δ (ppm):** 8.00 (m, 1H), 7.43 (dt, *J*=2.5, 8.5 Hz, 1H), 7.35 (dt, *J*=6.5, 8.5 Hz, 1H), 6.93-6.86 (m, 2H), 6.73 (d, *J*=8.5 Hz, 1H), 5.84 (s, 2H), 3.89 (s, 3H).

**^13^C-NMR (125.7 MHz; CDCl_3_) δ (ppm):** 168.5, 161.9 (dd, *J_CF_*=11.9, 248.1 Hz), 159.8 (dd, *J_CF_*=11.6, 249.3 Hz), 150.0, 134.7 (d, *J_CF_*=3.1 Hz), 131.6 (d, *J_CF_*=2.5 Hz), 130.9 (dd, *J_CF_*=5.0, 9.4 Hz), 124.8 (dd, *J_CF_*=3.9, 13.5 Hz), 123.0, 116.9, 111.5 (dd, *J_CF_*= 3.9, 21.1 Hz), 110.8, 104.4 (dd, *J_CF_*=25.4, 26.8 Hz), 51.8.

**ESI-MS (ESI^+^) m/z** 264 (M+H)^+^

**2’,4’-difluoro-4-amino-3-iodo-[1,1’]-biphenyl-3-carboxylic acid methyl ester (6b)**

**Formula:** C14H10F2INO2

**HPLC (GEN1) RT:** 20.92 min.

**^1^H-NMR (400 MHz; CD_3_COCD_3_) δ (ppm):** 8.05-8.04 (m, 2H), 7.54-7.50 (m, 1H), 7.12-7.08 (m, 2H), 6.78 (s ample, 1H), 3.87 (s, 3H), 2.85 (d, *J*=16.5, 1H).

**^13^C-NMR (100.6 MHz; CD_3_COCD_3_) δ (ppm):** 168.1, 162.6 (dd, *J_CF_*=9.8, 250.1 Hz), 160.6 (dd, *J_CF_*=9.5, 250.8 Hz), 150.5 (d, *J_CF_*=6.2 Hz), 144.9 (d, *J_CF_*=2.6 Hz), 132.7 (d, *J_CF_*=2.5 Hz), 132.1 (dd, *J_CF_*=3.8, 7.6 Hz), 124.4, 124.2 (dd, *J_CF_*=3.1, 10.9 Hz), 112.6 (dd, *J_CF_*=2.9, 16.9 Hz), 111.1 (d, *J_CF_*=1.6 Hz), 104.9 (t, *J_CF_*=20.5), 86.2 (d, *J_CF_*= 11.8; C-I), 52.5.

**MS (ESI^+^) m/z** 390 (M+H)^+^

**4-Acetoxy-2’,4’-difluoro-[1,1’]-biphenyl-3-carboxylic acid (7a)**

**Formula:** C_15_ H_12_ O_4_ F_2_

**HPLC** (GEN 1) RT: 6.28 min.

**^1^H-NMR** (500 MHz, acetone-d^6^): δ(ppm): 8.18 (s,1H), 7.8 (dd, *J*=8.0,2.0Hz,1H), 7.62 (dd, *J*=8.5,15.0Hz, 1H), 7.3 (d,*J*=8.5 Hz,1H), 7.15-7.13 (m, 2H), 2.29 (s, CH_3_).

**^13^C-NMR**  (125.7 MHz, acetone-d^6^): δ (ppm): 169.4, 165.2,162.9 (*J*_CF_=12.2, 248.2 Hz), 160.0 ( *J*_CF_=11.9, 250.4 Hz), 151.1, 134.9 (d, *J*_CF_=3.0 Hz), 133.4 (d, *J*_CF_=1.4 Hz), 132.7(dd, *J*_CF_=3.0,8.0 Hz), 125.2, 124.3 (dd, *J*_CF_= 3.9,13.5 Hz), 112.3 (dd, *J*_CF_=3.6, 20.9 Hz), 104.6 (t, *J*_CF_ =26.1 Hz), 21.5.

**IR (cm^-1^):** ν max. 3078 (ν_OH_), 1767(ester, ν_C=O_),1698 (acid,ν_C=0_)

**MALDI-TOF-MS**: M+Na^+^ : 314.86 m/z=292.05(M^+^,100%)

**4-Acetoxy-2’,4’-difluoro-5-iodo-[1,1’]-biphenyl-3-carboxylic acid (7b)**

**Formula:** C_15_ H_11_ O_4_ F_2_ I

**HPLC** (GEN1) TR: 12.43 min.

**^1^H-NMR** (500 MHz, acetone-d^6^): δ (ppm): 8.27 (s,1H), 8.19 (s,1H), 7.69 (dd, *J*=8.5,15.0Hz, 1H), 7.17-7.13 (m, 2H), 2.35 (s, 3H, OCH_3_).

**^13^C-NMR**  (100.56 MHz, acetone-d^6^): δ (ppm): 172.1, 163.3 (*J*_CF_=10.1, 295.8 Hz), 161.3, 160.5 ( *J*_CF_=11.9, 250.4 Hz), 145.8, 132.6 (dd, *J*_CF_=4.5, 9.1Hz), 131.8 , 128.4, 113.7, 112.3 (dd, *J*_CF_=3.8, 17.6 Hz) 105.1 (t, *J*_CF_ =25.94 Hz), 85.6, 20.5.

**MALDI-TOF-MS**: MH^+^ : 419.07 (antracene and 0.1% TFA) m/z=417.95 (M^+^,100%)

***N*-[5-(2,4-difluorophenyl)salicyloyl]-glycine or *N*-[2’,4’-difluoro-4-hydroxy-[1,1’]-biphenyl-3-carbonyl]-glycine (8a)**

**Formula:** C_15_ H_11_ NO_4_ F_2_

**Yield: 45%**

**HPLC** (GEN 1) RT: 6.20 min.

**^1^H-NMR** (500 MHz, acetone-d^6^): δ (ppm): 8.01 (s,1H), 7.61 (d, *J*=8.5,1H), 7.53 (dd, *J*=9.0,15.5Hz, 1H), 7.09(dd, *J*=2.5,13.5Hz, 2H), 7.02 (d, *J*= 8.5Hz,1H), 4.2 (d, *J*= 5.5Hz,2H).

**^13^C-NMR**  (125.7 MHz, acetone-d^6^): δ (ppm): 171.0, 170.9, 162.9 (*J*_CF_=11.8, 259.1Hz), 161.96, 160.4 ( *J*_CF_=12.1, 248.6 Hz), 135.5 (d, *J*_CF_= 3.2 Hz), 132.5 (dd, *J*_CF_=4.9, 9.7 Hz), 128.2 (d, *J*_CF_ =2.1Hz), 126.2, 125.4 (dd, *J*_CF_= 3.9,13.5 Hz), 118.9, 115.3, 112.5 (dd, *J*_CF_=3.6, 20.9 Hz), 104.9 (t, *J*_CF_ =26.1 Hz), 41.5

**FAB-MS:** MH^+^: 308.15 M/Z= 307,06 (M^+^,100%)

***N*-[5-(2,4-difluorophenyl)-3-iodosalicyloyl]-glycine or *N*-[2’-,4’-difluoro-4-hydroxy-5-iodo-[1,1’]-biphenyl-3-carbonyl]-glycine (8b)**

Formula: C_15_ H_10_ NO_4_ F_2_ I

**Yield: 94%**

**HPLC** (GEN1) RT: 10.74 min.

**^1^H- NMR** (500 MHz, acetone-d^6^): δ (ppm): 8.11 (s,1H), 8.10 (s,1H), 7.59 (dd, *J*=9.5,15.1Hz, 1H), 7.16-7.09 (m,2H), 4.19 (d, *J*=6.0 Hz,2H).

**^13^C-NMR** (100.56 MHz, acetone-d^6^): δ (ppm): 170.8, 170.5, 163.3 (*J*_CF_=11.9, 247.7Hz), 161.22, 159.9 (*J*_CF_=12.3, 261.1Hz), 144.5 (d, *J*_CF_= 3.5 Hz), 132.6 (dd, *J*_CF_=4.7, 9.8 Hz), 128.3, 127.9, 123.9 (d, *J*_CF_= 13.6 Hz), 114.9, 112.6 (dd, *J*_CF_=3.4, 21.5 Hz), 105.01 (t, *J*_CF_ =26.2Hz), 41.6

**MALDI-TOF-MS:** M^+^:431.80 (α-cyano-4-hydroxycinnamic acid) m/z =432.96 (M^+^.100%)

***N*-[5-(2,4-difluorophenyl)salicyloyl]-L-leucine *tert*-butyl ester or *N*-[2’,4’-difluoro-4-hydroxy-[1,1’]-biphenyl-3-carbony]-L-leucine *tert*-butyl ester (13a)**

**Formula:** C_23_H_27_F_2_NO_4_

**Yield: 55%**

**HPLC** (GEN 1) RT: 20.79 min.

**^1^H-NMR** (300 MHz, CDCl_3_): δ (ppm): 12.3 (s,1H), 7.54-7.48 (m,1H), 7.35 (dd, *J*=8.7,15.3Hz, 1H), 7.03-6.86 (m,3H), 4.72 (d,*J*=5.4 Hz,2H), 1.75-1.6 (m,3H), 1.46 (s, 9H), 0.97 (d, *J*=2.4 Hz,3H), 0.95 (d,*J*=2.4,3H).

**^13^C-NMR**  (125.7 MHz, CDCl_3_): δ (ppm): 172.3, 169.4, 163.1 (dd, *J*_CF_ =11.7, 228.3 Hz), 161.9, 160.4(dd, *J*_CF_ =11.9, 248.63 Hz), 134.9(d, *J*_CF_ =2.9 Hz), 131.3 (dd, *J*_CF_= 9.7, 4.9 Hz), 128.2, 126.2, 125.4 (d, *J*_CF_ = 14.1 Hz), 118.9, 115.3, 112.5 (dd, *J*_CF_=3.64, 20.9 Hz), 104.9 (t, *J*_CF_ =26.1 Hz), 82.8, 51.5, 41.8, 28.1, 25.2, 22.9, 22.2.

**MALDI-TOF-MS:** [M-^t^Bu]^+^: 364.08 (α-cyano-4-hydroxycinnamic acid) m/z=419.19 (M^+^,100%)

***N*-[5-(2,4-difluorophenyl)-3-iodo-salicyloyl]-L-leucine *tert*-butyl ester or *N*-[2’-,4’-difluoro-4-hydroxy-5-iodo-[1,1’]-biphenyl-3-carbonyl)-L-leucine *tert*-butyl ester (13b)**

**Formula:** C_23_H_26_F_2_NO_4_I

**Yield: 95%**

**HPLC** (GEN 1) RT: 27.56 min.

**^1^H-NMR** (500 MHz, CDCl_3_): δ (ppm): 7.92 (s,1H), 7.56 (s,1H), 7.37(dd, *J*=9.0,15.5Hz, 1H), 6.96 (td, *J*=8.0, 2.0 Hz, 1H), 6.84 (td, *J*=8.5,2.5 Hz, 1H), 4.67 (d,*J*=5.0 Hz.,1H), 1.75-1.6 (m,3H), 1.46 (s, 9H), 0.95 (t, *J*= 6.5 Hz,4H).

**^13^C-NMR** (125.7 MHz, CDCl_3_): δ (ppm): 173.4, 169.3, 162.4 (dd, *J*_CF_ =11.7, 249.5 Hz), 160.1, 159.5 (dd, *J*_CF_ =11.9, 249.9 Hz), 143.9, 131.3 (dd, *J*_CF_ = 9.4, 4.6 Hz), 127.1, 126.4 (d, *J*_CF_ =3.1 Hz), 123.1 (d, *J*_CF_ = 9.8 Hz), 113.4, 111.8 (dd, *J*_CF_=3.9, 20.9 Hz), 104.4 (t, *J*_CF_ =26.3 Hz), 87.5, 83.1, 51.9, 41.1, 28.1, 25.2, 22.9, 21.9.

**MALDI-TOF-MS**: MH^+^ :546.23 (α-cyano-4-hydroxycinnamic acid) m/z=545,08 (M^+^,100%)

***N*-[5-(2,4-difluorophenyl)salicyloyl]-L-leucine or *N*-[2’,4’-difluoro-4-hydroxy-[1,1’]-biphenyl-3-carbonyl]-L-leucine (12a)**

### Formula: C_19_H_19_F_2_NO_4_

**MW:** 363,35

**HPLC** (GEN 1) RT: 11.15 min.

**^1^H-NMR** (500 MHz, CDCl_3_): δ (ppm): 10.6 (s,COOH), 7.63(s,1H), 7.5 (d, *J*=8.5Hz, 1H), 7.31 (dd, *J*=8.5,15.0Hz, 1H), 7.01(d, *J*=8.5 Hz, 1H), 6.92-6.83 (m,2H), 4.84-4.80 (m,1H), 1.82-1.75 (m,3H), 0.97 (t, *J*=5.0 Hz,6H)

**^13^C-NMR** (125.7 MHz, acetone-d^6^): δ (ppm): 173.8, 170.9, 162.3 (dd, *J*_CF_ =11.7, 249.3 Hz), 161.9, 160.4 (dd, *J*_CF_ =11.7, 248.7 Hz), 135.5 (d, *J*_CF_ =2.9 Hz), 132.6 (dd, *J*_CF_ = 9.7, 4.9 Hz), 128.3, 126.2, 125.2 (d, *J*_CF_ = 14.1 Hz), 118.9, 115.3, 112.5 (dd, *J*_CF_=3.6, 21.4 Hz), 104.9 (t, *J*_CF_ =26.64 Hz), 51.6, 40.8, 25.2, 22.9, 21.6

**ESI-MS**: MH^+^ : 364.2 m/z= 363.2 (M^+^, 100%)

.

***N*-[5-(2,4-difluorophenyl)-3-iodosalicyloyl]-L-leucine or *N*-[2’,4’-difluoro-4-hydroxy-5-iodo-[1,1’]-biphenyl-3-carbonyl]-L-leucine (12b)**

**Formula:** C_19_H_18_F_2_NO_4_I

**Yield: 86%**

**HPLC** (GEN 1) RT: 18.38 min.

**^1^H-NMR** (500 MHz, CDCl_3_): δ (ppm): 8.01 (s,1H), 7.5 (s,1H), 7.33 (dd, *J*=9.0,15.0 Hz,1H), 6.96-6.86 (m,3H), 4.85-4.80 (m,1H), 1.82-1.69 (m,2H), 1.57 (s,1H), 0.97 (d, *J*=5.5 Hz,6H)

**^13^C-NMR** (125.7 MHz, acetone-d^6^): δ (ppm): 173.5, 170.7, 161.7 (dd, *J*_CF_ =11.7, 249.4 Hz), 159.2, 158.6 (dd, *J*_CF_ =11.7, 248.6 Hz), 144.6 (d, *J*_CF_ =3.0 Hz.), 132.7 (t, *J*_CF_ = 5.6 Hz), 128.4, 127.9, 115.3, 114.9, 112.6 (d, *J*_CF_ =17.7 Hz), 105.1 (t, *J*_CF_ =26.5 Hz), 86.8, 51.9, 40.6, 25.6, 23.3, 21.6.

**ESI-MS**: MH^+^ :490.1 m/z= 489.1 (M^+^,100%)

***N*-[5-(2,4-difluorophenyl)salicyloyl]-L-alanine ethyl ester or *N*-[2’,4’-difluoro-4-hydroxy-[1,1’]-biphenyl-3-carbonyl]-L-alanine ethyl ester (11a)**

**Formula:** C_18_H_17_F_2_NO_4_

**Yield:** 55%

**HPLC** (GEN 1) RT: 11.49 min.

**^1^H- NMR** (300 MHz, CDCl_3_): δ (ppm): 7.52 (s,1H), 7.48 (s, 1H), 7.33 (d, *J*=9.6Hz, 1H), 7.05-6.89 (m, 3H), 4.73 (t,*J*=10.5 Hz,1H), 4.24 (dd,*J*=21.3,10.5 Hz, 2H), 0.29 (d, *J*=10.5 Hz,3H), 0.06 (t,*J*=10.8 Hz,3H)

**^13^C- NMR**  (75.4 MHz, CDCl_3_): δ (ppm): 173.1, 169.2, 161.2, 134.9, 131.1 (dd, *J*_CF_= 9.3, 4.7 Hz), 126.1 (d, *J*_CF_ =2.7 Hz), 125.6, 124.3, 118.8, 113.8, 111.49 (d, *J*_CF_=3.8Hz), 104.2 (t, *J*_CF_=26.14 Hz), 61.9, 48.3, 18.4, 14.1.

**MALDI-TOF-MS**: MH^+^ : 350.09 (α-cyano-4-hydroxycinnamic acid) m/z 349,11(M^+^,100%)

***N*-[5-(2,4-difluorophenyl)-3-iodo-salicyloyl]-L-alanine ethyl ester or *N*-[2’,4’-difluoro-4-hydroxy-5-iodo-[1,1’]-biphenyl-3-carbonyl]-L-alanine ethyl ester (11b)**

**Formula:** C_18_H_16_F_2_NO_4_I

**Yield:**  98%

**HPLC** (GEN 1) RT: 17.49 min.

**^1^H- NMR** (300 MHz, CDCl_3_): δ (ppm): 7.99 (s,1H), 7.52 (s, 1H), 7.48 (s,1H), 7.33 (dd, *J*=15.0, 9.0,1H), 7.97-6.86 (m,2H), 4.72 (t,J=6.9 Hz,1H), 4.24 (dd,*J*=14.4, 7.2Hz, 2H), 1.52 (d, *J*=7.2 Hz,3H), 1.30 (t,*J*=7.2 Hz,3H)

**13C- NMR**  (75.4 MHz, CDCl_3_ ): δ (ppm): 172.9, 168.5, 159.9, 143.9, 131.2, 127.2, 126.3, 113.6, 111.9, 104.5, 87.2, 62.1, 48.6, 18.3, 14.1.

**MALDI-TOF-MS**: MH^+^:476.05 (α-cyano-4-hydroxycinnamic acid) m/z =475,00 (M^+^,100%)

***N*-[5-(2,4-difluorophenyl)salicyloyl]-L-alanine methyl ester or *N*-[2’,4’-difluoro-4-hydroxy-[1,1’]-biphenyl-3-carbonyl]-L-alanine methyl ester (10a)**

**Formula:** C_17_H_15_F_2_NO_4_

**Yield:** 30%

**HPLC** (GEN1) RT: 12.08 min.

**^1^H- NMR** (300 MHz, CDCl_3_): δ (ppm): 8.04 (s,1H), 7.73 (s,1H), 7.5 (t, *J*=8.5 Hz,1H), 7.3-7.0 (m, 2H), 6.91-6.58 (m, 2H), 4.46 (t,*J*=7.2 Hz,1H), 3.90 (dd,*J*=14.1, 7.2 Hz, 1H), 3.38 (s,3H), 1.8 (s,3H)

**^13^C- NMR**  (75.4 MHz, CDCl_3_ ): δ (ppm): 172.9, 168.3, 164.3, 161.7, 146.8, 137.4, 131.1 (dd, *J*_CF_ = 9.34, 4.67 Hz), 126.1 (d, *J*_CF_ =2.71 Hz), 125.6, 124.3, 118.8, 113.8, 111.49 (d, *J*_CF_=3.84Hz), 104.2 (t, *J*=26.1 Hz), 61.9, 48.3, 18.4

**MALDI-TOF-MS**: MH^+^: 336.14 (acid α-cyano-4-hydroxycinnamic acid) m/z: 335,09 (M^+^, 100%)

**Éster metílico de *N*-[5-(2,4-difluorophenyl)-3-iodosalicyloyl]-L-alanine methyl ester or *N*-[2’,4’-difluoro-4-hydroxy-5-iodo-[1,1’]-biphenyl-3-carbonyl] -L-alanine methyl ester (10b)**

**Formula:** C_17_H_14_F_2_NO_4_I

**Yield:** 97%

**HPLC** (GEN1) RT: 18.37 min.

**^1^H- NMR** (300 MHz, CDCl_3_): δ (ppm): 8.59 (s,1H), 7.96 (s,1H), 7.59 (s,1H), 7.33-7.27 (m, 1H), 6.91-6.58 (m, 2H), 4.75 (t,*J*=6.0 Hz.1H), 3.90 (dd,*J*=14.1, 7.2 Hz, 1H), 3.79 (s,3H), 1.52 (d, *J*_CF_ = 9.0 Hz ,3H)

**^13^C- NMR**  (75.4 MHz, CDCl_3_): δ (ppm): 173.7, 168.8, 159.8, 149.3, 143.8,136.4, 131.2, 127.1, 126.4, 123.9, 113.4, 111.67 (d, *J*_CF_= 17.5 Hz) 104.3 (t, *J*_CF_ =26.1 Hz) 52.8, 48.4, 17.8

**MALDI-TOF-MS**: MH^+^:461.96 (α-cyano-4-hydroxycinnamic acid) m/z= 460,99 (M^+^,100%)

***N*-[5-(2,4-difluorophenyl)salicyloyl]-L-alanine or *N*-[2’,4’-difluoro-4-hydroxy-[1,1’]-biphenyl-3-carbonyl]-L-alanine (9a)**

### Formula: C_16_H_13_F_2_NO_4_

**Yield:** 76%

**HPLC** (GEN1) RT: 6.11 min

**^1^H-NMR** (500 MHz, CD_3_OD): δ (ppm): 8.04 (d,*J*=1.5 Hz,1H), 7.53 (d, *J*=8.5 Hz,1H), 7.5 (dd, *J*=8.5, 15 Hz,1H), 7.01 (dd, *J*=9.1,8.5 Hz, 3H), 4.63 (t,*J*=7.5,15 Hz.1H), 1.52 (d, *J*=7.0 Hz.,3H)

**^13^C-NMR** (125.7 MHz, CD_3_OD ): δ (ppm): 170.12, 169.7, 163.6 (d, *J*_CF_=12.1, 247.4 Hz), 161.1(d, *J*_CF_=11.9, 248.7 Hz), 160.3, 135.31 (d, *J*_CF_ =3.4 Hz), 132.6 (dd, *J*_CF_ =9.4, 4.7), 130.2, 127.3, 125.8 (dd, *J*_CF_ = 3.8, 13.61 Hz), 118.8,117.3, 112.6 (dd, *J*_CF_=3.7, 21.5Hz), 105.0 (t, *J*_CF_ =25.6 Hz), 34.7, 17.8

**MALDI-TOF-MS**: MH^+^:319.93 (α-cyano-4-hydroxycinnamic acid) m/z: 321,08 (M^+^,100%)

***N*-[5-(2,4-difluorophenyl)-3-iodosalicyloyl]-L-alanine or *N*-[2’,4’-difluoro-4-hydroxy-5-iodo-hydroxy-[1,1’]-biphenyl-3-carbonyl]-L-alanine (9b)**

### Formula: C_16_H_12_F_2_NO_4_I

**Yield:** 95%

**HPLC** (GEN1) RT: 11.13 min.

**^1^H- NMR** (500 MHz, CDCl_3_): δ (ppm): 7.94(s,1H), 7.58 (s,1H), 7.31 (dd, *J* =7.5, 2.5Hz,1H), 6.87 (dt, *J*=91, 39.5 Hz, 2H), 4.74 (t,*J*=7.0 Hz.1H), 1.53 (d, *J*=7.5 Hz.,3H)

**^13^C-NMR**  (125.7 MHz, CDCl_3_): δ (ppm): 176.7, 168.9, 162.4 (d, *J*_CF_=11.9, 247.4 Hz), 159.7,159.5 (d, *J*_CF_=11.7, 249.9 Hz), 143.9,131.2 (dd, *J*_CF_ =9.4, 4.5), 127.3, 126.6, 122.7 (dd, *J*_CF_ =3.6, 13.4 Hz), 113.6, 111.8 (dd, *J*_CF_=3.9, 21.3Hz), 104.4 (t, *J*_CF_ =25.6 Hz), 87.1, 29.4, 17.8

**MALDI-TOF-MS**: MH^+^ : 445.87 (α-cyano-4-hydroxycinnamic acid) m/z=446,97(M^+^,100%)

***N*-[5-(2,4-difluorophenyl)salicyloyl]-β-alanine methyl ester or *N*-[2’,4’-difluoro-4-hydroxy-[1,1’]-biphenyl-3-carbonyl]-β-alanine methyl ester (23a)**

### Formula: C_17_H_15_F_2_NO_4_

**Yield:** 90%

**HPLC** (GEN1) RT: 10.91 min

**^1^H-NMR** (300 MHz, CDCl_3_): δ (ppm): 12.3 (s,1H), 7.47-7.45 (m,2H), 7.31-7.24 (m,1H), 7.01 (s,1H), 6.89-6.86 (m, 2H), 3.68 (t, *J*=6.3 Hz., 2H), 3.67 (s,3H), 2.64 ( (t,*J*=5.7 Hz.2H)

**^13^C-NMR**  (75.4 MHz, CDCl_3_ ): δ (ppm): 173.3, 169.7, 162.5 (dd, *J*_CF_ =11.9, 190.7Hz), 161.1, 159.1 (dd, *J*=11.6, 191.4 Hz), 134.6, 130.9 (dd, J_CF_=9.2, 4.8 Hz ), 126.0, 125.9,124.0(d,*J*_CF_ =13.3 Hz), 118.7, 114.2, 111.6 (dd, *J*_CF_=3.9, 21.2Hz), 104.3(t, *J*_CF_ =26.4Hz), 51.9, 34.9, 33.3.

**MALDI-TOF-MS:** MH^+^: 336,12 (α-cyano-4-hydroxycinnamic acid) m/z= 335,09 (M^+^,100%)

***N*-[5-(2,4-difluorophenyl)-3-iodosalicyloyl]-β-alanine methyl ester or *N*-[2’-,4’-difluoro-4-hydroxy-5-iodo-[1,1’]-biphenyl-3-carbonyl]-β-alanine methyl ester (23b)**

**Formula:** C_17_H_14_F_2_NO_4_I

**Yield:** 90%

**HPLC** (GEN1) RT: 17.69 min.

**^1^H-NMR** (300 MHz, CDCl_3_): δ (ppm): 7.98 (dd,*J*=1.5, 2.1 Hz,1H), 7.48 (t, *J*=1.5 Hz, 1H), 7.34-7.30 (m,2H), 7.02-6.86 (m, 2H), 3.72 (t, *J*=6.0 Hz., 2H), 3.71 (s,3H), 2.68 ( (t,*J*=6.0 Hz.2H)

**^13^C-NMR**  (75.4 MHz, CDCl_3_ ): δ (ppm): 173.3, 169.1, 161.5 (dd, *J*_CF_ =11.9, 190.7Hz), 160.7, 160.1 (dd, *J*=11.6, 191.4 Hz), 143.7, 131.0 (dd, *J*_CF_=9.5, 4.8 Hz), 127.1, 126.2,114.1, 111.7 (t, *J*_CF_=17.4Hz), 104.5(t, *J*_CF_ =26.2 Hz), 87.1,52.1, 35.3, 33.2.

**MALDI-TOF:** MH^+^: 461.93 (α-cyano-4-hydroxycinnamic acid and 0.1% TFA) m/z= 460,99 (M^+^,100%)

***N*-[5-(2,4-difluorophenyl)salicyloyl]-β-alanine or *N*-[2’-,4’-difluoro-4-hydroxy-[1,1’]-biphenyl-3-carbonyl] ]-β-alanine (22a)**

**Formula:** C_16_H_13_F_2_NO_4_

**Yield:** 90%

**HPLC** (GEN1) RT: 6.89 min.

**^1^H-NMR** (500 MHz, CD_3_OD): δ (ppm): 7.94 (dd,*J*=1.0, 2.5 Hz,1H), 7.53 (dt, *J*=2.0, 8.5 Hz, 1H), 7.51-7.45 (m,1H), 7.03-6.97 (m, 3H), 3.67 (t, *J*=7.0 Hz., 2H), 2.63 ( (dd,*J*=6.5, 10.5 Hz.2H)

**^13^C-NMR** (125.7 MHz, CD_3_OD): δ (ppm): 175.5, 170.3, 163.5(dd, *J*_CF_ =11.3, 247.3 Hz), 160.9 (dd, *J*_CF_ = 11.7, 248.7 Hz), 160.4, 135.2 d, *J*_CF_ =3.4 Hz), 132.6 (dd, *J*_CF_ =4.7, 9.4 Hz),129.7, 127.2, 125.8 (t, *J*_CF_=9.7 Hz ), 118.5,117.3, 112.5 (dd, J_CF_=3.9, 21.6Hz), 105.1(t, *J*=27.01 Hz), 36.5, 34.6.

**MALDI-TOF:** MH^+^: 319.98 (α-cyano-4-hydroxycinnamic acid and 0.1% TFA) m/z: 321.08 (M^+^,100%)

***N*-[5-(2,4-difluorophenyl)-3-iodosalicyloyl]-β-alanine or *N*-[2’-,4’-difluoro-4-hydroxy-5-iodo-[1,1’]-biphenyl-3-carbonyl]- β-alanine (22b).**

**Formula:** C_16_H_12_F_2_NO_4_I

**Yield:** 96%

**HPLC** (GEN1) RT: 12.11 min.

**^1^H-NMR** (500 MHz, CD_3_OD): δ (ppm): 7.99 (t,*J*=1.5,1H) 7.86 (s, 1H) 7.50-7.45 (m,1H) 7.05-7.0 (m, 2H) 3.64 (t, *J*=6.5 Hz., 2H) 2.65 ( t,*J*=7.5 Hz,2H)

**^13^C-NMR** (125.7 MHz, CD_3_OD): δ (ppm): 175.3, 170.9, 163.8 (dd, *J*_CF_ =12.23, 248.12 Hz), 161.2, 161.0 (dd, *J*_CF_ = 12.1, 249.1 Hz), 144.6 (d, *J*_CF_ =3.9 Hz), 132.7 (dd, *J*_CF_ =4.7, 9.4 Hz), 128.4 (d, *J*_CF_ = 10.8 Hz), 124.5 (t, *J*_CF_=13.7 Hz), 115.7, 112.7 (dd, *J*_CF_=3.9, 21.5Hz), 105.1(t, *J*_CF_ =26.7 Hz), 86.7, 36.8, 34.4.

**MALDI-TOF-MS:** MH^+^ : 447,92 (α-cyano-4-hydroxycinnamic acid and 0.1% TFA) m/= 446,97 (M^+^,100%)

***N*-[5-(2,4-difluorophenyl)salicyloyl]-L-serine methyl ester or *N*-[2’,4’-difluoro-4-hydroxy-[1,1’]-biphenyl-3-carbonyl]-L-serine methyl ester (15a)**

**Formula:** C_17_H_15_F_2_NO_5_

**Yield:** 55%

**HPLC** (GEN1) RT: 5.41 min.

**1H-NMR** (300 MHz, CDCl_3_): δ (ppm): 7.60 (s,1H) 7.49 (t, *J*=2.1, 6.9 Hz,1H), 7.39-7.24 (m,1H), 7.02 (d, *J*=7.8, 1H), 6.88-6.86 (m, 2H), 4.84 (t, *J*=3.6 Hz, 1H), 4.11-4.05 ( m,2H), 3.80 (s, 3H)

**13C-NMR**  (75.4 MHz, CDCl_3_): δ (ppm): 170.8, 169.85, 161, 135.2, 131.1,126.9,126.4, 125.7, 118.7,116.4, 113.8,111.62, 104.4, 62.9, 54.7, 53.1

***N*-[5-(2,4-difluorophenyl)-3-iodosalicyloyl]-L-serine methyl ester or *N*-[2’,4’-difluoro-4-hydroxy-5-iodo-[1,1’]-biphenyl-3-carbonyl]-L-serine methyl ester (15b)**

**Formula:** C_17_H_14_F_2_NO_5_I

**Yield:** 41%

**HPLC** (GEN1) RT: 10.88 min.

**^1^H- NMR** (300 MHz, CD_3_OD): δ (ppm): 8.05 (s,1H), 8.03 (t, *J*=2.1, 6.9 Hz,1H), 7.54-7.52 (m,1H) 7.09-7.03 (m, 2H), 4.78 (t, *J*=4.5 Hz, 1H), 3.98 ( t, *J*=5.4 Hz, 2H), 3.78 (s, 3H)

**^13^C-NMR** (75.4 MHz, CD_3_OD ): δ (ppm): 172.0, 171.1, 161.1, 145.0, 132.7,129.1,128.6, 127.7, 125.6, 115.6,112.7(d, *J*_CF_ =21.2Hz), 105.2 (t, *J*_CF_ =25.8 Hz), 86.7, 62.5, 56.7, 52.9

**MALDI-TOF-MS:** M+Na: 493.06 (α-cyano-4-hydroxycinnamic acid).

***N*-[5-(2,4-difluorophenyl)salicyloyl]-L-serine or *N*-[2’,4’-difluoro-4-hydroxy-[1,1’]-biphenyl-3-carbonyl)]-L-serine (14a)**

**Formula:** C_16_H_13_F_2_NO_5_

**Yield:** 56%

**HPLC** (GEN1) RT: 3.65 min.

**^1^H-NMR** (300 MHz, CD_3_OD): δ (ppm): 8.07 (s,1H) 7.55-7.49 (m, 5H) 4.72 (s, 1H) 4.01 (ddd, *J*=2.4, 6.6, 22.2,2H)

**^13^C-NMR** (125.7 MHz, CD_3_OD ): δ (ppm): 169.3, 163.6 (dd, *J*_CF_ =11.9, 247.5), 161.5 (dd, *J*_CF_ =11.9, 247.5), 159.72, 135.2 (d, *J*_CF_ =3.5 Hz), 132.6 (dd, *J*_CF_ =4.7, 9.4 Hz), 130.7, 127.4, 125.8

(d, *J*_CF_=13.8 Hz), 118.3, 117.9, 112.6 (dd, *J*_CF_ =3.7, 21.1Hz), 105.1 (t, *J*_CF_ =25.9 Hz), 63.0, 56.7

**MALDI-TOF-MS:** MH^+^:338.02 (α-cyano-4-hydroxycinnamic acid and 0.1% TFA) m/z=337,07(M^+^,100%)

***N*-[5-(2,4-difluorophenyl)-3-iodosalicyloyl]-L-serine *N*-[2’,4’-difluoro-4-hydroxy-5-iodo-[1,1’]-biphenyl-3-carbonyl]–L-serine (14b)**

**Formula:** C_16_H_12_F_2_NO_5_I

**Yield:** 33%

**HPLC** (GEN1) RT: 7.20 min.

**^1^H- NMR** (500 MHz, CD_3_OD): δ (ppm): 8.55 (d, *J*=.5,1H), 8.02 (d,*J*=7.0,1H), 7.46 (t, *J*=8.5 Hz,2H), 7.03 (t, *J*=8.5 Hz, 1H), 4.72 (t, *J*=1.2Hz, 1H), 3.99 (t, *J*= 5.5,2 H )

**^13^C-NMR**  (100.5 MHz, CDCl_3_ ): δ (ppm): 173.4, 169.4, 163.9 (dd, *J*_CF_ =16.2, 284.8 Hz), 159.4, 150.2 (dd, *J*_CF_ =16.2, 286.3 Hz), 144.2, 131.2 (d, *J*_CF_ =9.7 Hz), 127.5, 127.3 (d, *J*_CF_ =17.9 Hz), 113.6, 112.0 (d, *J*_CF_ =20.9 Hz), 104.5 (t, *J*_CF_ =26.0 Hz), 87.2, 62.6, 54.7

**ES-MS:** M^+^ : 477,97 m/z= 477,99 (M^+^,100%)

***N*-[5-(2,4-difluorophenyl)salicyloyl]-L-aspartic α-methyl ester and β-*tert*-butyl ester or *N*-[2-,4’-difluoro-4-hydroxy-[1,1’]-biphenyl-3-carbonyl]-L-aspartic α-methyl ester and β-*tert*-butyl ester (19a)**

**Formula**: C_22_H_23_F_2_NO_6_

**Yield:** 47%

**HPLC** (GEN1) RT: 12.73 min.

**^1^H-NMR** ( 500 MHz, CDCl_3_): δ (ppm): 7.54 (d, *J*=8.0,1H), 7.26 (s,1H), 7.06 (t, *J*=8.5 Hz,1H), 6.89 (dd, *J*=8.5, 14.5 Hz, 1H), 6.58 (d, *J*=8.5 Hz, 1H), 6.48-6.40 (m,2 H ), 4.64 (q, *J*=3.0, 5.0 Hz, 1H), 3.35 (s, 3H), 2.54 (ddd, *J*=5.5, 17.0, 58.5 Hz, 2H), 1.01 (s, 9H)

**^13^C-NMR**  (125.7 MHz, CD_3_OD ): δ (ppm): 171.3, 170.1,169.1, 162.2 (dd, *J*_CF_ =11.9, 249.0 Hz), 161.3, 159.4 (dd, *J*_CF_ =11.8, 249.0), 1354.7 (d, *J*_CF_=2.2 Hz), 130.9 (dd, J_CF_ =4.6, 9.5 Hz), 126.7 (d, *J*_CF_ =3.01 Hz), 125.6, 123.9 (dd, *J*_CF_ =3.9,13.7 Hz), 118.4, 114.1, 111.5 (dd, *J*_CF_ = 3.9, 21.3Hz), 104.2 (dd, *J*_CF_ =26.8, 25.4 Hz), 82.1, 52.7, 49.1, 37.3, 27.7

**MALDI-TOF-MS:** M+Na^+^ : 458.11 (3,5-dihydroxybenzoic acid) m/z= 435,14 (M^+^,100%)

***N*-[5-(2,4-difluorophenyl)-3-iodosalicyloyl]-L-aspartic α-methyl ester and β-*tert*-butyl ester or *N*-[2’,4’-difluoro-4-hydroxy-5-iodo-[1,1’]-biphenyl-3-carbonyl]-L-aspartic α-methyl ester and β-*tert*-butyl ester (19b)**

**Formula**: C_22_H_22_F_2_NO_6_I

**Yield:** 90%

**HPLC** (GEN1) RT: 19.64 min

**^1^H- NMR** ( 500 MHz, CDCl_3_): δ (ppm): 8.02(s,1H), 7.67 (s,1H), 7.58 (s,1H), 7.33 (s, 1H), 6.95-6.89 (m,2H), 5.01 (s, 1H), 3.80 (s, 3H), 2.96 (dd, *J*=16.0, 85.5 Hz, 2H), 1.44 (s, 9H)

**^13^C- NMR**  (125.7 MHz, CD_3_OD): δ (ppm): 170.9, 170.2,168.8, 162.5 (dd, *J*=11.9, 249.0 Hz), 160.1, 159.6 (dd, *J*=11.7, 250.6) 144.1, 131.1 (dd, *J*=4.6, 9.7 Hz), 127.4, 126.6, 125.8, 122.8 (dd, *J*=14.0 Hz), 113.8, 111.9 (d, J= 21.4Hz), 104.6 (t, J=26.0 Hz), 87.1, 82.4, 53.1, 49.1, 37.2, 28.0.

**MALDI-TOF-MS:** MH^+^: 562.10 (α-cyano-4-hydroxycinnamic acid) m/z= 561,046 (M^+^,100%)

***N*-[5-(2,4-difluorophenyl)salicyloyl]-L-aspartic β-*tert*-butyl ester or *N*-[2’,4’-difluoro-4-hydroxy-[1,1’]-biphenyl-3-carbonyl]-L-aspartic β-*tert*-butyl ester (18a)**

**Formula:** C_21_H_21_F_2_NO_6_

**Yield:** 56%

**HPLC** (GEN1) RT: 8.85 min.

**^1^H- NMR** ( 300 MHz, CD_3_OD): δ (ppm): 7.53 (s,1H), 7.4-7.24 (m,2H), 7.05-6.81 (m,3H), 5.01-4.98 (m, 1H), 2.96 (ddd, *J*=6.6, 25.8, 60.3 Hz, 2H), 1.42 (s, 9H)

**^13^C-NMR**  (125.7 MHz, CDCl_3_ ): δ (ppm): 172.9, 171.6, 167.2 ,162.0 (dd, *J*_CF_ =11.8, 248.7 Hz), 160.6, 159.5 (dd, *J*_CF_ =14.4, 250.0 Hz), 134.7, 130.9 (dd, *J*_CF_ =4.6, 9.4 Hz), 126.7, 125.6, 123.9 (dd, *J*_CF_ =3.6,13.3 Hz), 118.4, 114.1, 111.4 (dd, *J*_CF_ = 3.4, 21.11Hz) 104.2 (t, *J*_CF_ =25.6 Hz) 82.1, 48.8, 37.4, 28.1.

***N*-[5-(2,4-difluorophenyl)-3-iodosalicyloyl]-L-aspartic β-*tert*-butyl ester or *N*-[2’,4’-difluoro-4-hydroxy-5-iodo-[1,1’]-biphenyl-3-carbonyl]-L-aspartic β-*tert*-butyl ester (18b)**

**Formula:** C_21_H_20_F_2_NO_6_I

**Yield:** 94%

**HPLC** (GEN1) RT: 15.32 min.

**^1^H- NMR** ( 300 MHz, CDCl_3_): δ (ppm): 8.63 (dd, *J*=1.8, 6.3 Hz,1H), 7.99 (s,1H), 7.44-7.4 (m,2H), 6.94-6.84 (m,2H), 4.92 (m, 1H), 2.99 (dd,*J*=12.3,47.4 Hz, 2H), 1.41 (s, 9H)

**^13^C- NMR**  (125.7 MHz, CDCl_3_ ): δ (ppm): 173.4, 170.4,168.6, 160.1, 147.4, 138.3, 127.2, 126.5, 124.5, 114.1, 111.3 ,104.2 , 86.9, 81.8, 49.5, 37.3, 27.9.

**MALDI-TOF-MS:** MH^+^: 546.10 (α-cyano-4-hydroxycinnamic acid and 0.1 % TFA) m/z: 547,03 (M^+^,100%)

***N*-[5-(2,4-difluorophenyl)salicyloyl]-L-aspartic α-methyl ester or *N*-[2’,4’-difluoro-4-hydroxy-[1,1’]-biphenyl-3-carbonyl]-L-aspartic α-methyl ester (17a)**

**Formula:** C_18_H_15_F_2_NO_6_

**Yield:** 78%

**HPLC** (GEN1) RT: 5.46 min.

**^1^H-NMR** ( 500 MHz, CDCl_3_): δ (ppm): 7.52 (s,1H) 7.23 (s,1H) 7.05 (t, *J*_CF_ =8.5 Hz,1H), 7.03 (dd, J_CF_ =8.5, 14.6 Hz, 1H), 7.03 (d, *J*=9.4 Hz, 1H), 6.86-6.84 (m,2 H ), 5.01 (q, *J*=4.5, 12.0 Hz, 1H), 3.78 (s, 3H), 3.10 (dd, *J*_CF_ =4.6, 26.4 Hz, 2H)

**^13^C-NMR**  (125.7 MHz, CDCl_3_): δ (ppm): 175.9, 170.8,169.4, 162.5 (dd, *J*_CF_ =11.7, 248.4 Hz), 161.1, 159.6 (dd, *J*_CF_ =11.5, 249.8 Hz), 135.2 (d, *J*_CF_ =2.3 Hz), 131.1 (dd, J_CF_ =4.5, 9.5 Hz), 126.2 (d, J_CF_ =2.9 Hz), 125.8, 123.9,118.8, 113.7, 111.52 (dd, *J*_CF_ = 3.79, 21.43Hz), 104.4 (t, *J*_CF_ =25.7Hz), 53.2, 48.8, 35.7

***N*-[5-(2,4-difluorophenyl)-3-iodosalicyloyl]-L-aspartic α-methyl ester or *N*-[2’,4’-difluoro-4-hydroxy-5-iodo-[1,1’]-biphenyl-3-carbonyl]-L-aspartic α-methyl ester (17b)**

**Formula:** C_18_H_14_F_2_NO_6_I

**Yield:** 96%

**HPLC** (GEN1) RT: 11.52 min.

**^1^H-NMR** ( 500 MHz, acetone-d^6^): δ(ppm): 8.11 (s,1H), 8.00 (s,1H), 7.58-7.53 (m,1H), 7.18-7.10 (m, 2H), 5.11-5.07 (m,1H), 3.72 (s, 3H), 3.02 (ddd, *J*=5.0, 20.0, 35.0 Hz, 2H)

**^13^C-NMR**  (125.7 MHz, CD_3_OD ): δ (ppm): 171.2, 170.7,169.8, 161.2 (dd, *J*_CF_ =11.6, 249.3 Hz), 160.6, 159.8 (dd, *J*_CF_ =11.8, 247.1 Hz), 144.2, 132.1 (dd, J_CF_ =4.6, 9.6 Hz), 127.8,127.4, 123.4,114.2, 111.9 (dd, J_CF_ = 17.6Hz), 104.4 (t, J_CF_ =26.0 Hz), 86.3, 52.3, 48.8, 35.3.

**MALDI-TOF-MS:** MH^+^: 506.02 (3,5-dihydroxybenzoic acid and 0.1% TFA) m/z: 504,98 (M^+^,100)

***N*-[5-(2,4-difluorophenyl)salicyloyl]-L-aspartic or *N*-[2’,4’-difluoro-4-hydroxy-[1,1’]-biphenyl-3-carbonyl]-L-aspartic (16a)**

**Formula:** C_17_H_13_F_2_NO_6_

**Yield:** 46%

**HPLC (**GEN1) RT: 3.88 min.

**^1^H-NMR** (500 MHz, acetone-d^6^): δ (ppm): 8.67 (d, *J*=8.0,1H), 7.98 (s,1H), 7.61 (dt, *J*=2.0,9.0 Hz,1H), 7.54-7.50 (m,1H), 7.13-7.03 (m,1H), 7.03 (d, *J*=8.5,1H), 5.10 (q, *J*=7.5, 5.5 Hz, 1H), 3.03 (ddd, *J*=5.0, 17.0, 43.0 Hz, 2H)

**^13^C-NMR**  (125.7 MHz, acetone-d^6^): δ (ppm): 172.2, 172.1,170.4, 163.1 (dd, *J*_CF_ =12.2, 247.2 Hz), 161.8, 160.4 (dd, *J*_CF_ =11.8, 248.3 Hz), 135.6 (d, *J*_CF_ =2.9 Hz), 132.5 (dd, *J*_CF_ =4.8, 9.7 Hz), 128.4 (d, *J*_CF_ =2.1 Hz), 126.4, 125.4 (dd, *J*_CF_ =3.9,13.7 Hz), 118.9, 115.5, 112.5 (dd, *J*_CF_ = 3.6, 21.2 Hz) 104.5 (t, *J*_CF_ =25.9 Hz) 49.9, 36.1

**MALDI-TOF-MS:** MH^+^ :363.92 (α-cyano-4-hydroxycinnamic acid) m/z: 365,07 (M^+^,100%)

***N*-[5-(2,4-difluorophenyl)-3-iodosalicyloyl]-L-aspartic or *N*-[2’-,4’-difluoro-4-hydroxy-5-iodo-[1,1’]-biphenyl-3-carbonyl]-L-aspartic (16b)**

### Formula: C_17_H_12_F_2_NO_6_I

**Yield:** 97%

**HPLC** (GEN1) RT: 7.53 min

**^1^H- NMR** ( 500 MHz, CD_3_OD ): δ (ppm): 8.03 (d, *J*=1.5 Hz,1H), 7.94 (s,1H), 7.52-7.47 (m ,1H), 7.06-7.02 (m,2H), 5.0 (q, *J*=7.5, 5.0 Hz, 1H), 2.98 (ddd, *J*=5.5, 17.0, 54.0 Hz, 2H)

**^13^C-NMR**  (100.6 MHz, CD_3_OD): δ (ppm): 174.1, 173.8,170.8, 162.4 (dd, *J*_CF_ =12.1, 247.5 Hz), 161.1, 160.0 (dd, *J*_CF_ =12.1, 249.5), 144.9 (d, *J*_CF_ =3.3 Hz), 132.7 (dd, *J*_CF_ =3.4, 7.6 Hz) 128.8,128.5, 124.5 (dd, *J*_CF_ =10.9 Hz), 115.6, 112.7 (dd, *J*_CF_ = 3.5, 17.0Hz), 105.2 (t, *J*_CF_ =20.6 Hz) 83.3, 50.7, 36.5

**MALDI-TOF-MS:** MH^+^ : 489.74 (α-cyano-4-hydroxycinnamic acid) m/z=490,96 (M^+^,100%)

***N*-[5-(2,4-difluorophenyl)salicyloyl]-L-asparagine *tert*-butyl ester or *N*-[2’,4’-difluoro-4-hydroxy-[1,1’]-biphenyl-3-carbonyl]-L-asparagine *tert*-butyl ester (21a)**

**Formula:** C_21_H_22_F_2_N_2_O_5_

**Yield:** 17%

**HPLC** (GEN1) RT: 8.33 min

**^1^H-NMR** ( 500 MHz, DMSO ): δ (ppm): 7.99 (d, *J*=8.0 Hz,1H), 7.59 (s,1H), 7.49 (d, *J*=8.5 Hz,1H), 7.43-7.26 (m,1H), 7.01 (d, *J*=9.0 Hz, 1H), 6.91-6.83 (m, 2H), 4.86-4.84 (m,1H), 2.92 (ddd, *J*=4.5, 16.0, 57.0 Hz, 2H), 1.47 (s, 9H)

**^13^C- NMR**  (125.7 MHz, CD_3_OD): δ (ppm): 175.1, 171.5,169.2, 163.6 (dd, *J*_CF_ =11.9, 247.5 Hz) 161.0 (dd, *J*_CF_ =12.1, 248.7), 159.82, 135.3 (d, *J*_CF_ =3.5 Hz), 132.6 (dd, *J*_CF_ =4.7, 9.8 Hz), 130.5,128.3,127.4, 125.7 (dd, *J*_CF_ =4.2, 13.6 Hz), 118.4, 117.7, 112.6 (dd, *J*_CF_ = 3.9, 21.4 Hz), 105.1 (t, J_CF_=26.78 Hz), 83.3, 51.5, 37.7, 28.2.

**MALDI-TOF-MS:** M^+^-^t^BuO: 365.1 (α-cyano-4-hydroxycinnamic acid) m/z: 420,14 (M^+^,100)

***N*-[5-(2,4-difluorophenyl)-3-iodosalicyloyl]-L-asparagine *tert*-butyl ester or *N*-[2’,4’-difluoro-4-hydroxy-5-iodo-[1,1’]-biphenyl-3-carbonyl]-L-asparagine *tert*-butyl ester (21b)**

**Formula:** C_21_H_21_F_2_N_2_O_5_I

**Yield:** 53%

**HPLC** (GEN1) RT: 13.92 min.

**^1^H-NMR** ( 500 MHz, CDCl_3_ ): δ (ppm): 8.03 (d, *J*=7.5 Hz, 1H), 7.98 (s,1H), 7.57 (s,1H), 7.30-7.29 (m,1H), 6.92-6.84 (m, 2H), 4.86-4.84 (m,1H), 2.92 (ddd, *J*=4.5, 16.0, 57.0 Hz, 2H), 1.46 (s, 9H)

**^13^C-NMR** (125.7 MHz, CDCl_3_): δ (ppm): 172.5, 169.5,168.9, 163.6, 161.0, 169.1, 144.1, 131.1, 127, 126.7,113.9, 111.9 (d, J_CF_ = 21.45Hz) 105.6, 86.9, 83.3, 49.9, 36.5, 27.9.

**MALDI-TOF-MS:** MH^+^: 544.79 (α-cyano-4-hydroxycinnamic acid) m/z: 546,04 (M^+^,100%)

***N*-[5-(2,4-difluorophenyl)salicyloyl]-L-asparagine or *N*-[2’,4’-difluoro-4-hydroxy-[1,1’]-biphenyl-3-carbonyl]-L-asparagine (20a)**

**Formula:** C_17_H_14_F_2_N_2_O_5_

**Yield:** 54%

**HPLC** (GEN1) RT: 3.45 min.

***1*H- NMR** ( 500 MHz, CD_3_OD ): δ (ppm): 8.00 (s,1H), 7.54-7.44 (m,2H), 7.02-6.98 (m, 3H), 4.99-4.97 (m,1H), 2.98-2.88 (m, 2H)

***13*C- NMR**  (125.7 MHz, CD_3_OD): δ (ppm): 175.2, 174.3,169.4, 163.6 (dd, *J*_CF_ =11.9, 247.5 Hz) 159.9, 160.95 (dd, *J*_CF_ =12.3, 248.7 Hz), 135.3 (d, *J*_CF_ =3.54 Hz), 132.6 (dd, *J*_CF_ =4.6, 9.3Hz), 130.5 (d, *J*_CF_ =1.8 Hz),128.3,127.4, 125.8 (dd, *J*_CF_ =4.0, 13.7Hz), 118.4 (*J*_CF_=8.6Hz), 117.6, 112.6 (dd, *J*_CF_ = 3.8, 21.7 Hz), 111.5, 105.1 (t, *J*_CF_ =26.7 Hz), 50.7, 37.6.

**MALDI-TOF-MS:** M^+^ : 363.97 (3,5-dihydroxybenzoic acid and 0.1 %TFA) m/z: 364,08 (M^+^,100%)

***N*-[5-(2,4-difluorophenyl)-3-iodosalicyloyl]-L-asparagine or *N*-[2’-,4’-difluoro-4-hydroxy-5-iodo-[1,1’]-biphenyl-3-carbonyl]-L-asparagine (20b)**

**Formula:** C_17_H_13_F_2_N_2_O_5_I

**Yield:** 67%

**HPLC** (GEN 1) RT: 5.93 min.

**^1^H- NMR** ( 400 MHz, acetone-d^6^): δ (ppm): 8.38 (s, 1H) 8.27 (s,1H) 7.87-7.81 (m,2H) 7.41-7.37 (m,1H) 5.03 (m,1H) 3.0 (s, 2H)

**^13^C- NMR**  (100.6 MHz, acetone-d^6^): δ (ppm): 175.1, 174.4, 170.6, 163.8 (dd, *J*_CF_ =12.13,248.6 Hz), 161.0, 160.9 (dd, *J*_CF_ =12.2, 249.6 Hz), 144.8, 132.6 (t, *J*_CF_=10.1 Hz), 128.5 (d, *J*_CF_ =15.1 Hz), 127.9, 127.1, 124.3 (d, *J*_CF_ =13.0 Hz), 115.5, 112.7 (d, *J*_CF_ = 25.2Hz), 105.1 (t, *J*_CF_ = 26.1 Hz), 86.7, 49.6, 37.27.

**MALDI-TOF-MS:** MH^+^: 490.87 (α-cyano-4-hydroxycinnamic acid) m/z= 489,98 (M^+^, 100%)

***In vitro* fibril inhibition*.***

The mutant Y78F TTR protein (0.4 mg.mL^‑1^) was incubated for 30 min (pH 7.6, 37ºC) with 6 concentrations of test compounds ranging from 0 to 40 μM in 96‑well microplates. The pH was lowered to the optimal value for fribrillization (pH = 4.2, and incubation at 37^0^C was extended to 1.5 h with shaking ([[5]](#endnote-3)). The absorbance at 340 nm was recorded at 1 min intervals for each well. Time course curves were obtained from which initial rates of fibril formation correspond to the slope of the linear increase of absorbance. In turn, plots of these initial rates versus test compounds concentrations follow exponential equation (equation 1) from which different parameters related to the potency of a compound can be derived.

**V_0_ = A + B e ^–C [I]^**

Equation 1

IC50% value is the inhibitor concentration at which the initial rate of fibril formation is half of that in the absence of inhibitor. RA(%) is the percentage of reduction in fibril formation rate induced by a given concentration of test compound relative to the rate of experiments at zero concentration of test compound. Values of RA(%) of 100% indicate that the inhibitor is able to fully prevent fibril formation (Figure S1).

**RA (%) = 100·A/(A+B)**

***T4 Competition assays***

Recombinant wild type TTR (30 nM) , produced in an *E. coli* expression system and isolated according to Almeida et al. (1997), was incubated overnight at 4ºC with a trace amount of ^125^I‑T_4_ (specific activity 1250 µCi/µg, concentration 320 µCi/mL) (Perkin Elmer, Boston MA) in the presence of increasing amounts of test compounds (0‑10 μM). Protein‑bound ^125^I‑T_4_ was separated from free ^125^I‑T_4_ by gel filtration on a BioGel P6‑DG (Bio‑Rad) column. Competition curves were obtained by plotting the ratio T_4_ bound/total T_4_ against the logarithm of inhibitor concentration from which values of EC_50_ were derived. When these T_4_ displacement data was referred to the EC_50_ of cold T_4_ using the ratio: EC_50_ of T_4_/EC_50_ of test compound, the relative T_4_ displacement potencies for each inhibitor were obtained (Figure S2).

***Assay binding of selectivity***

Whole human plasma (5 µL) was incubated for 1 hour at room temperature with labeled T_4_ (^125^I‑T_4_) and with each compound at 10 mM concentration (2 µL). Subsequently the proteins were separated by native polyacrylamide gel electrophoresis ([[6]](#endnote-4)). T_4_ displacement was detected by phosphorimaging of the dried gel (Figure S3).

**Stabilizing effect of inhibitors on TTR denaturation kinetics in urea by circular dichroism.**

Procedure for far UV CD measurements:

Stock solutions in ethanol (9 mM) of the inhibitors **1a** (diflunisal or DIF) and **1b** (iododiflunisal or IDIF) were prepared. Protein stock solutions of wtTTR and TTRY78F of 1 mg/ml of protein in 50 mM of sodium phosphate buffer, 1mM EDTA, pH 7.0 were also prepared from mother solutions of both proteins containing 4 mg/mL (72.7 μM) of wtTTR and 3 mg/mL (55.5 μM) of TTRY78F that were determined by spectrophotometrical methods. To 2 mL of both wtTTR and TTRY78F stock solutions, 8 μl of either ethanol (control) or the ethanolic solutions of **1a** and **1b** were added. The samples were incubated overnight at 25ºC. Next, to these same samples, 6 mL of a 10M urea solution in 50 mM sodium phosphate buffer pH 7.0 and 2 mL of sodium phosphate buffer 50 mM pH 7.0 were added as to reach final concentrations in the samples of 6M urea, 3.6 μM of protein and 7.2 μM of inhibitor. The samples were incubated over a period of 200 hours at 25ºC and far-UV CD spectra (210-250 nm) were periodically recorded in a JASCO 600 instrument. The spectra were processed using the standard program J 850 for Windows.

Procedure for near-UV CD measurements:

In a parallel set of experiments, samples were prepared to measure CD spectra in the range of 250-320 nm were changes in absorptions from the aromatic residues of the protein (Tyr, Phe and Trp) can be observed. Similar estabilizing effects of the proteins due to the presence of the inhibitors could be recorded. However, such changes could not be properly quantified since at these wavelengths the aromatic groups of the inhibitors also absorbed. The experimental procedure used was as follows.

Both **1a** and **1b** were dissolved in ethanol to prepare 46 mM stock solutions. From them, 26 μl of each were taken and added to 1.6 mL of a wtTTR stock solution of 4 mg/mL (74 μM) in 50 mM of sodium phosphate buffer, 1 mM EDTA, pH 7.0, that were further diluted by adding 400 μl of sodium phosphate buffer, 50 mM, pH 7.0. A similar procedure was followed for the TTRY78F protein. Thus, 26 μl of the inhibitors stock solutions were added to 722 μl of a TTRY78F stock solution of 9 mg/mL (163 μM) in 50 mM of sodium phosphate buffer, 1 mM EDTA, pH 7.0, that were further diluted by adding 1278 μl of sodium phosphate buffer, 50 mM, pH 7.0. Control samples were also prepared that contained the same volume of ethanol but not the inhibitors. The samples were incubated overnight at 25ºC. Next, to this same samples, 3 mL of a 10 M urea solution in 50 mM sodium phosphate buffer, pH 7.0 were added as to reach final concentrations in the samples of 6M urea, 24 μM of protein and 47 μM of inhibitor. The samples were incubated over a period of 200 hours at 25ºC and far-UV CD spectra (250-320 nm) were periodically recorded in a JASCO-600 instrument using quartz cuvettes of 0.05 cm of light path.

Raw spectral data was processed using the J‑850 for Windows Standard Analysis. From the different sets of CD spectra, frac­tion unfolded (Fu) data was calculated, normalized and plotted using the FigP programme which is presented as Figure 9 in the main text.

In figure 9 the rates of TTRwt and TTRY78F tetramer dissociation in 6M urea are seen to proceed at different rates. Denaturation of proteins up to 80% is reached after 24 and 48 h for TTRY78F and TTRwt, respectively. Indeed, these values were modified by adding diflunisal and more dramatically by iododiflunisal. Thus, in figure 9a, it can be observed that TTRwt (3.6 μM) was com­pletely denatured after 200 h in 6M urea. In contrast, protein denaturation after addition of a 7.2 μM concentracion of iododi­flunisal, is reduced to 30%. In the same conditions diflunisal was much less able to prevent tetramer dissociation and 60% of unfolding was observed after 200 h. Similar patterns were record­ed (figure 9b) when using TTRY78F (3.6 μM) which was totally unfolded after 200 h in 6M urea. Thus, while a 7.2 μM concentra­tion of iododiflunisal leads to a 30% denaturation after 200 h, the same concentration of diflunisal causes 80% of tetramer dissociation after 200 h, thus, confirming the higher stabilizing properties of iododiflunisal over diflunisal.

***Computational evidences supporting the iodination hypothesis:GRID studies of TTR’s binding site.***

Affinity grids were used to energetically describe the iodine affinity for the six pockets (HBPs) described in the seminal work of Blake and coworkers ([[7]](#endnote-5)). To calculate the affinity grid the protein was embedded within a 3D grid and then a probe atom was placed successively at each grid point. The interaction energy between the probe and the protein was evaluated and recorded. The result is a 3D volume that described the binding of ligand atoms within the site. By contouring the maps at appropiate levels, it was easy to identify binding “hotspots” of favorable interaction. The starting point of the computational analysis was the protein structure of the complex of TTR with T4 (PDB code 2ROX). As a first step, hydrogen atoms were added to the complex and then the geometry of the system was optimized keeping fixed all the non-hydrogen atoms. The calculations were carried out within the molecular mechanics framework using the all-atom force field of Cornell et al ([[8]](#endnote-6)) as implemented in AMBER6 ([[9]](#endnote-7)), with a distance-dependent dielectric constant of 1r and a 12 Å cutoff for non-bonded interactions. Affinity maps were calculated for iodine atom probe, as well as for the rest of halogens (fluorine, chlorine and bromine) for comparison purposes, by means of GRID software ([[10]](#endnote-8)), version 22. The analysis was carried in a box of 44×40×30 Å^3^ centered in the channel of the TTR, after removing ligands, counterions, water molecules, and using a grid spacing of 0.5Å. The obtained maps were analyzed with MINIM and FILMAP modules of GRID in order to find interaction energy minima. By contouring the maps at appropiate levels, favorable interaction zones were identified. Visual inspection of the maps was done with the GRID module of InsightII software ([[11]](#endnote-9)). Because in the crystal structures the two binding sites are symmetry-related, the final analysis was only done on the A-A’ binding site.

***Molecular modeling studies on TTR complexes*** *with iododiflunisal and its betaAlaOMe* (**23b**) *and betaAlaOH* (**22b**) *conjugates and their comparison with* ***T4****-TTR complex.*

Protein structures of the complexes of TTR with the three iododiflunisal derivatives (PDB codes 1Y1D for iododiflunisal, 3FC8 for the iododiflunisal betaAlaOMe conjugate (**23b**) and 3FCB for the betaAlaOH conjugate (**22b**) and T_4_ (PDB code 2ROX) were analysed. Protein complexes were pre-treated using the same protocol as in the affinity grid studies: hydrogen atoms were added to the complex, the geometry of the system was optimized keeping fixed all the non-hydrogen atoms and the most stable binding site was choose for further calculations. Protein-ligand interactions were analyzed with the ANAL (hydrogen bonds) and CARNAL (hydrophobic interactions) modules of AMBER. The GRID program (Error: Reference source not found) was used to analyze the binding site of TTR in order to assess if halogens are located in favorable positions, therefore iodine and fluorine probes were used. The protocol and parameters used in this study were the same as described in the GRID studies of TTR binding site section.

***Protein complex preparation and crystallization*.**

Recombinant transthyretin was expressed in *Escherichia coli* and isolated and purified as previously reported (Error: Reference source not found). The protein was dialyzed against 10 mM HEPES buffer (pH 7.5) and concentrated to 11 mg/ml. The protein solutions were then incubated for 24 h with a 10-fold-molar excess of diflunisal analog compounds at 20 ºC. Crystals of the complexes were obtained by hanging-drop vapor-diffusion techniques at 14ºC. For the TTR:iododiflunisal-betaAlaOMe (**23b**) complex, 2 μl of the protein:iododiflunisal-betaAlaOMe solution were mixed with 2 μl of the reservoir solution containing 2.0M ammonium sulphate, 7% glycerol and 0.2 M sodium acetate pH 5.2 as precipitant. In the case of the TTR:iododiflunisal-betaAlaOH complex (**22b**), crystals suitable for X-ray diffraction were obtained with the following reservoir solutions: 2.2 M ammonium sulphate, 7% glycerol and 0.2 M sodium acetate pH 5.4. Crystals for data collection were transferred to reservoir solutions containing increasing concentrations of glycerol (10-20%) and flash frozen to liquid nitrogen.

*Data collection, processing and refinement.*

X-ray diffraction data were collected at 100 K using synchrotron radiation on beamlines ID14-EH1 (λ=0.934 Å) of the European Synchrotron Radiation Facility (ESRF, Grenoble, France). For each crystallographic data set, the crystal orientation and integration of the reflections was performed with *MOSFLM* ([[12]](#endnote-10)) while scaling and merging of the reflections were performed using programs SCALA and TRUNCATE ([[13]](#endnote-11)). Details of the crystallographic data collection are presented in **Table S1**.

The structures of the two complexes were determined by molecular replacement with Phaser ([[14]](#endnote-12)) using the atomic coordinates of T119M-TTR (PDB code 1F86) ([[15]](#endnote-13)), after removal of water molecules and the mutation of residue 119 to threonine. Three cycles of automated refinement using the program CNS ([[16]](#endnote-14)) alternated with manual model building with the graphic program Turbo-FRODO were performed ([[17]](#endnote-15)). Water molecules were added manually at the position of positive peaks (>3σ) on the difference Fourier maps where good hydrogen bond geometry existed. The refinement was monitored using R_free_, calculated from a set of 5% of the reflections, which were not used in the refinement. For each TTR complex data, the (2*F*_o_- *F*_c_) and (*F*_o_- *F*_c_) electron-density maps clearly showed positive electron density in the two hormone-binding sites corresponding to the position of the diflunisal derivatives. The atomic coordinates of the binding compounds were obtained from the HIC-UP database ([[18]](#endnote-16)) and were manually fitted into the density. The model was further refined with REFMAC ([[19]](#endnote-17)) using the CCP4i program suite (Error: Reference source not found). Half-occupancy was given to the diflunisal derivatives as they are located on the twofold crystallographic symmetry axis. The quality of the final model was checked using program PROCHECK ([[20]](#endnote-18)). Relevant refinement statistics are presented in **Table S1**.

**Table S1**. Data collection and refinement statistics

|  | TTR:iododiflunisal-betaAlaOMe (**23b**) | TTR:iododiflunisal-betaAlaOH (**22b**) |
| --- | --- | --- |
| **Data collection** |  |  |
| Space Group | P2_1_2_1_2 | P2_1_2_1_2 |
| Unit Cell dimensions (Å) | a=42.2 b=85.1 c=63.1 | a=43.0 b=85.7 c=63.6 |
| Resolution range (Å) | 50.97-1.85 | 63.76-1.80 |
| No. of observations (total/unique) | 189703/ 20049 | 162528 / 22496 |
| Multiplicity (overall/last shell) | 9.5 / 6.8 | 7.2 / 7.4 |
| Rmerge (overall/last shell) | 5.6 / 23.4 | 5.8 / 16.0 |
| Completeness (%)(overall/last shell) | 99.8 / 99.2 | 99.9 / 98.7 |
| I/σ(I) (overall/last shell) | 32.4 / 8.2 | 27.0 / 9.9 |
| Mathews Coefficient (Å^3^Da^-1^) | 2.10 | 2.11 |
| Solvent content (%) | 41.0 | 41.1 |
| **Structure refinement** |  |  |
| Rfactor / Rfree | 19.9 / 21.5 | 19.9 / 23.0 |
| No. of unique reflections  (working / test set) | 18982 / 950 | 22499 / 1136 |
| Water molecules | 132 | 157 |
| Residues with alternate conformations | S115A | S85A, N27B |
| Total number of atoms | 1940 | 1987 |
| Average protein B-factor (Å^2^) | 15.3 | 16.7 |
| Average main-chain B-factor (Å^2^) | 13.8 | 15.2 |
| Average side-chain B-factor (Å^2^) | 17.1 | 18.2 |
| Average ligand B-factor (Å^2^) | 16.1 | 24.3 |
| R.m.s. bonded B’s (Å^2^) | 16.5 | 16.8 |
| R.m.s. deviations from ideal values  Bonds (Å)  Angles (º) | 0.017  1.3 | 0.018  1.4 |
| Ramachandran plot statistics  Most favoured regions (%)  Additionally allowed regions (%) | 92.5  7.5 | 91.0  9.0 |

**REFERENCES cited in the SUPPORTING INFORMATION:**

1. + These authors contributed equally to the work. [↑](#footnote-ref-2)
2. + [↑](#footnote-ref-3)
3. To whom correspondance should be addressed at: Unit of Glycoconjugate Chemistry, Institut de Química Avançada de Catalunya I.Q.A.C.-C.S.I.C., Jordi Girona 18-26, 08034 Barcelona (Spain). Ph: +34934006113, fax +34932045904, e-mail: gvpqbp@iiqab.csic.es. [↑](#footnote-ref-4)
4. . a) Barluenga J, González JM, García‑Martín MA, Campos PJ, Asensio G (1992) An expe­ditious and general aromatic iodination procedure. J Chem Soc Chem Commun 14:1016‑1017; b) Barluenga J, González, JM (1999). In: Scolastico C, Nicotra F, editors. Current Trends in Organic Synthesis eds. New York: Kluwer Academic / Plenum Publishers. pp 145-151; (b) Barluenga J (1999) Recent advances in selective organic synthesis mediated by transition metal complexes. Pure Appl. Chem. 71: 431-436. [↑](#endnote-ref-2)
5. . Dolado I, Nieto J, Saraiva MJ, Arsequell G., Valencia G, Planas A (2005) Kinetic assay for high‑throughput screening of *in vitro* transthyretin amyloid fibrillogenesis inhibitors. J Comb Chem 7: 246‑252. [↑](#endnote-ref-3)
6. . a) Almeida MR, Damas AM, Lans MC, Brouwer A, Saraiva, M.J. (1997) Thyroxine bind­ing to transthyretin Met 119. Comparative studies of different heterozygotic carriers and struc­tural analysis. *Endocrine* 6: 309‑315. b) Almeida MR, Macedo B, Cardoso I, Alves I, Valencia G, Arsequell G, Planas A, Saraiva MJ. (2004) Selective binding to transthyretin and tetramer stabilization in serum from patients with familial amyloidotic polyneuropathy by an iodinated diflunisal derivative. Biochem J 381: 351‑356. c) Lans MC, Klasson‑Wehler E, Willemsen M, Meussen E, Safe S, Brouwer A. (1993) Structure‑dependent, competitive interaction of hydroxy-polychlorobiphenyls ‑dibenzo‑p‑diox­ins and ‑dibenzofurans with human transthyretin. Chem Biol Interact 88: 7‑21. [↑](#endnote-ref-4)
7. . De la Paz, P, Burridge JM, Oatley SJ, Blake CCF (1992) Multiple modes of binding of thyroid hormones and other iodothyronines to human plasma transthyretin. In: Beddell CR, editor. The Design of Drugs to Macromolecular Targets. Chinchester : Wiley. pp 119-172. [↑](#endnote-ref-5)
8. . Cornell WD, Cieplak P, Bayly CI, Gould IR, Merz KM, Ferguson DM, Spellmeyer DC, Fox T, Caldwell JW, Kollman PA (1995) A second generation force field for the simulation fo proteins, nucleic acids, and organic molecules*.* J Am Chem Soc 117: 5179-5197. [↑](#endnote-ref-6)
9. . Case DA, Pearlman DA, Caldwell JW et al, (1999) AMBER Version 6. University of California, San Francisco. [↑](#endnote-ref-7)
10. . Goodford PJ (1985) A computational procedure for determining energetic favourable binding site of biological important macromolecules. J Med Chem 28: 849-857.

    [↑](#endnote-ref-8)
11. . Insight II, Version 2000. San Diego: Simulations IM; 2000. [↑](#endnote-ref-9)
12. . Leslie AGW (1992). In: Moras D, Podjarny AD, Thierri JC, editors. Crystallographic Computing 5: From Chemistry to Biology. Oxford: Oxford University Press. pp. 50-61. [↑](#endnote-ref-10)
13. . Collaborative Computational Project, Number 4. The CCP4 suite: programs for protein crystallography. Acta Crystallogr D Biol Crystallogr. 1994; 50: 760-3. [↑](#endnote-ref-11)
14. . McCoy AJ, Grosse-Kunstleve RW, Storoni LC, Read RJ (2005) Likelihood-enhanced fast translation functions. Acta Crystallogr D Biol Crystallogr. 6: 458-64. [↑](#endnote-ref-12)
15. . Sebastiao MP, Lamzin V, Saraiva MJ, Damas AM (2001) Transthyretin stability as a key factor in amyloidogenesis: X-ray analysis at atomic resolution. J Mol Biol. 306: 733-44. [↑](#endnote-ref-13)
16. . Brunger AT, Adams PD, Rice LM (1998) Recent developments for the efficient crystallographic refinement of macromolecular structures. Curr Opin Struct Biol. 8: 606-11. [↑](#endnote-ref-14)
17. . Roussel A, Cambillau C (1991) TurboFRODO in Silicon Graphics Geometry. Partner Directory, Silicon Graphics, Mountain View, CA. [↑](#endnote-ref-15)
18. . Kleywegt GJ, Jones TA (1998) Databases in protein crystallography. Acta Crystallogr D Biol Crystallogr 54: 1119-31. [↑](#endnote-ref-16)
19. . Murshudov GN, Vagin AA, Dodson EJ (1997) Refinement of macromolecular structures by the maximum-likelihood method. Acta Crystallogr D Biol Crystallogr. 53: 240-55. [↑](#endnote-ref-17)
20. . Laskowski RA, MacArthur MW, Moss DS, Thornton JM PROCHECK – A program to check the stereochemical quality of protein structures. (1993) J Appl Crystall 6: 238-291. [↑](#endnote-ref-18)
